# Genomic and transcriptomic profiling of combined small-cell lung cancer through microdissection: unveiling the transformational pathway of mixed subtype

**DOI:** 10.1186/s12967-024-04968-4

**Published:** 2024-02-21

**Authors:** Wenjuan Ma, Ting Zhou, Mengmeng Song, Jiaqing Liu, Gang Chen, Jianhua Zhan, Liyan Ji, Fan Luo, Xuan Gao, Pansong Li, Xuefeng Xia, Yan Huang, Li Zhang

**Affiliations:** 1 Department of Medical Oncology, State Key Laboratory of Oncology in South China, Guangdong Provincial Clinical Research Center for Cancer, Sun Yat-Sen University Cancer Center, 651 Dongfeng Road East, Guangzhou, 510060 Guangdong People’s Republic of China; 2 Department of Intensive Care Unit, State Key Laboratory of Oncology in South China, Guangdong Provincial Clinical Research Center for Cancer, Sun Yat-Sen University Cancer Center, Guangzhou, 510060 Guangdong People’s Republic of China; 3 Geneplus-Beijing Institute, Beijing, 102206 People’s Republic of China; 4 Department of Experimental Research, State Key Laboratory of Oncology in South China, Guangdong Provincial Clinical Research Center for Cancer, Sun Yat-Sen University Cancer Center, Guangzhou, 510060 Guangdong People’s Republic of China

**Keywords:** cSCLC, Microdissection, Monoclonal origin, Tumor immune microenvironment, RB1, TP53, Transdifferentiation

## Abstract

**Background:**

Combined small-cell lung carcinoma (cSCLC) represents a rare subtype of SCLC, the mechanisms governing the evolution of cancer genomes and their impact on the tumor immune microenvironment (TIME) within distinct components of cSCLC remain elusive.

**Methods:**

Here, we conducted whole-exome and RNA sequencing on 32 samples from 16 cSCLC cases.

**Results:**

We found striking similarities between two components of cSCLC-LCC/LCNEC (SCLC combined with large-cell carcinoma/neuroendocrine) in terms of tumor mutation burden (TMB), tumor neoantigen burden (TNB), clonality structure, chromosomal instability (CIN), and low levels of immune cell infiltration. In contrast, the two components of cSCLC-ADC/SCC (SCLC combined with adenocarcinoma/squamous-cell carcinoma) exhibited a high level of tumor heterogeneity. Our investigation revealed that cSCLC originated from a monoclonal source, with two potential transformation modes: from SCLC to SCC (mode 1) and from ADC to SCLC (mode 2). Therefore, cSCLC might represent an intermediate state, potentially evolving into another histological tumor morphology through interactions between tumor and TIME surrounding it. Intriguingly, RB1 inactivation emerged as a factor influencing TIME heterogeneity in cSCLC, possibly through neoantigen depletion.

**Conclusions:**

Together, these findings delved into the clonal origin and TIME heterogeneity of different components in cSCLC, shedding new light on the evolutionary processes underlying this enigmatic subtype.

**Supplementary Information:**

The online version contains supplementary material available at 10.1186/s12967-024-04968-4.

## Background

Lung cancer stands as the foremost cause of cancer-related mortality worldwide [1, 2], with small-cell lung carcinoma (SCLC) accounting for approximately 15% to 20% of cases [3, 4]. Within the spectrum of SCLC, combined small-cell lung carcinoma (cSCLC) [5] emerges as a rare subtype, characterized by additional components encompassing various histological types of non-small-cell lung carcinoma (NSCLC), such as adenocarcinoma (ADC), squamous cell carcinoma (SCC), large-cell neuroendocrine carcinoma (LCNEC), and large-cell carcinoma (LCC).

Recent years have witnessed significant strides in elucidating the molecular and transcriptional profiles of cSCLC through advanced techniques like next-generation sequencing [6–8]. Existing evidence posits that lung adenocarcinoma (LUAD) originates from alveolar type 2 (AT2) cells in the distal lung region and bronchioalveolar stem cells (BASCs) [9–12]. SCC, on the other hand, is believed to originate from basal and club cells, as well as AT2 cells.

In a broader context, LCNEC and SCLC trace their origins to neuroendocrine cells within the lung epithelium [9], with SCLC potentially deriving from AT2 cells in the lung epithelium [13, 14]. Notably, LUAD harboring epidermal growth factor receptor (EGFR) mutations has been observed to undergo transformation into SCLC, exhibiting resistance to EGFR-TKIs [15, 16]. SCLC transdifferentiation can also manifest in anaplastic lymphoma kinase (ALK)-translocated NSCLC following ALK-TKI therapy [17], immune-checkpoint inhibitor treatment [18], and even spontaneously without intervention [19].

Despite these insights, the monoclonal or polyclonal origin of cSCLC remains unclear. Existing studies have alluded to the possibility that cSCLC components may stem from the same pluripotent single clone [20]. However, a comprehensive exploration of the underlying mechanisms and biological behaviors of different components within cSCLC is lacking. While previous studies have probed the genomics and transcriptome of cSCLC as a whole, the specific genomic features and tumor immune microenvironment (TIME) of its distinct components remain elusive.

In this investigation, we conducted whole exome sequencing (WES) and transcriptomic profiling analysis on laser-microdissected tissue specimens encompassing ADC, SCC, LCNEC, LCC, and SCLC. These specimens were obtained from a cohort of 16 patients diagnosed with cSCLC. The primary objectives were to analyze genomic alterations, explore the relationships among these diverse cSCLC components, and assess the interplay between evolving cancer and the TIME.

## Methods

### Sample details

Between 2010 and 2019, 16 registered patients with cSCLC underwent thoracic surgery at Sun Yat-Sen University Cancer Center. Two pathologists confirmed the diagnosis of cSCLC through immunohistochemistry. Among these 16 patients, three presented with SCLC combined with LCC components, four with SCLC combined with LCNEC components, two with SCLC combined with SCC components, and seven with SCLC combined with LUAD components. None of the patients received any systematic anticancer treatment. Microdissection was employed to separate the two components of cSCLC, resulting in the collection of 32 formalin-fixed, paraffin-embedded (FFPE) tumor tissues from 16 patients (with two tumor regions obtained from each patient). Paired peripheral blood samples were procured during the operation. The study protocol received approval from the institutional review committee of the Cancer Center at Sun Yat-Sen University Cancer Center. We adhered to all relevant ethical codes for research involving human participants and obtained written informed consent.

### External database

Mutation files for pure ADC and SCC of the lung were downloaded from The Cancer Genome Atlas (TCGA). Pure SCLC data were retrieved from the cBioPortal website (https://www.cbioportal.org/study/summary?id=sclc_ucologne_2015) [21], while combined and pure LCNEC data were obtained from a previously published article.

### Analytical methods

Detailed information regarding the analysis methods employed in this study can be found in the Additional file 1: Methods [22, 23, 24, 25].

## Results

### Clinicopathological characteristics of cSCLC

The clinicopathological characteristics of 16 patients with cSCLC are summarized in Additional file 2: Table S1. Histologically, among the NSCLC patients, seven (43.8%) were ADC, two (12.5%) were SCC, and seven (43.8%) were LCC, encompassing both LCC and LCNEC of the lung.

A comprehensive multi-omics analysis was conducted on 32 samples from 16 cases, involving both WES and RNA sequencing (RNA-seq) by performing tissue laser microdissection (Additional file 1: Figure S1A, B). Notably, samples from case P4 were excluded from the analysis due to DNA extraction failure in the control sample. The median depth of WES reached 169.5 X for tumors and 218 X for controls, ensuring robust data quality and coverage.

### Genomic profiling comparison in different components of cSCLC

**Fig. 1:**
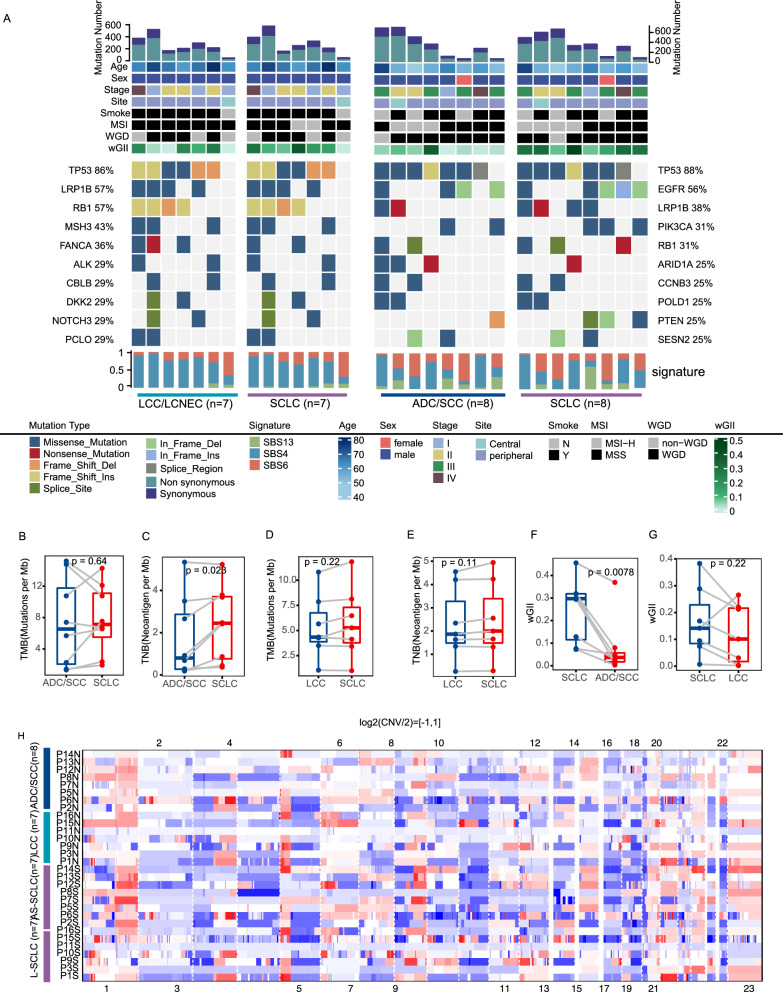
Genomic Landscape of cSCLC. **A** Top 10 recurrently mutated genes in cSCLC; Comparison of TMB **B** and TNB **C** between ADC/SCC and paired SCLC; Comparison of TMB **D** and TNB **E** between LCC and paired SCLC; **F** Comparison of CIN between SCLC and paired ADC/SCC; **G** Comparison of CIN between SCLC and paired LCC; **H** Copy number profile of all cSCLCs

All somatic single nucleotide variations (SNVs), insertions, and deletions are cataloged in Additional file 2: Table S2. Figure 1A and Additional file 2: Table S3 present the top 10 recurrently mutated genes. Key driver genes in cSCLC included TP53, RB1, EGFR, PI3CKA, and LRP1B. TP53 significantly co-occurred with RB1 (P = 0.041, odds ratio > 1) across the entire cohort. Notably, TP53 and RB1 emerged as the most recurrently mutated driver genes in the SCLC combined with the LCC/LCNEC (cSCLC-LCC/LCNEC) cohort, underscoring their pivotal roles in SCLC (Fig. 1A). In contrast, TP53 and EGFR were the top mutated driver genes in tumors with SCLC combined with ADC/SCC (cSCLC-ADC/SCC) (Fig. 1A). Other frequently mutated genes included LRP1B, MSH3, FANCA, and NOTCH3 in cSCLC-LCC/LCNEC, and LRP1B, PIK3CA, RB1, and ARID1A in cSCLC-ADC/SCC (Additional file 2: Table S4). Moreover, in terms of 30 samples, 30% (9/30) exhibited microsatellite instability-high (MSI-H), with the majority (67%, 6/9) being SCLC (Fig. 1A). COSMIC signatures [26] were identified in cSCLC, encompassing mutational signatures of tobacco exposure (SBS4), defective DNA mismatch repair with MSI (SBS6), and APOBEC cytidine deaminase activity (SBS13). However, no statistical differences were found among different components in cSCLC and in different cSCLC types (Fig. 1A, S2A–E).

Tumor mutation burden (TMB) showed no statistical difference (Fig. 1B, P = 0.64), while tumor neoantigen burden (TNB) in SCLC was significantly higher than that in paired ADC/SCC (Fig. 1C, P = 0.023; Additional file 2: Table S5). Both TMB and TNB exhibited no statistical difference between SCLC and paired LCC/LCNEC (Fig. 1D, E) and also between L-SCLC (SCLC of cSCLC-LCC/LCNEC) and AS-SCLC (SCLC of cSCLC-ADC/SCC) (Additional file 1: Figure S2F, G). At the cohort level, different tumor types showed no significant difference in both TMB and TNB (Additional file 1: Figure S2H, I).

The weighted genome instability index (wGII) for SCLC was higher than that of paired ADC/SCC (Fig. 1F, P = 0.0078), and the same trend was observed at the tumor-type level (Additional file 1: Figure S2J, P = 0.013). However, no statistical difference was observed between SCLC and paired LCC (Fig. 1G, P = 0.22), as well as between L-SCLC and AS-SCLC (Additional file 1: Fig. 2K). Notably, Copy number variation (CNV) profiles in SCLC revealed pronounced alterations (Fig. 1H). While no significant disparities were detected in whole-genome doubling (WGD) (Additional file 1: Figure S2L, M).

**Fig. 2:**
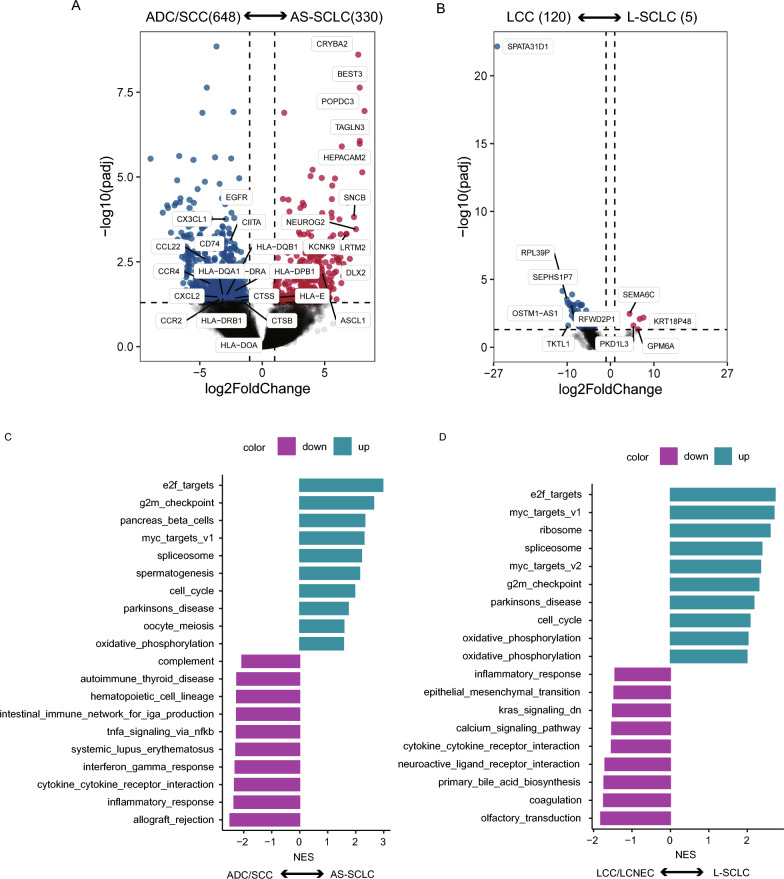
Transcriptome Profile in cSCLC. **A** Volcano plot of DEGs between SCLC and ADC/SCC groups; **B** Volcano plot of DEGs between SCLC and LCC groups; **C** GSEA analysis based on the pre-ranked gene set by log2FC between AS-SCLC and ADC/SCC groups; **D** GSEA analysis based on the pre-ranked gene set by log2FC between L-SCLC and LCC/LCNEC groups

Frequent TP53 CNV loss occurred in cSCLC (43%, 13/30), 4/8 in ADC/SCC, and 1/7 in LCC/LCNEC, particularly in ADC/SCC (50%, 4/8) and the AS-SCLC component (88%, 7/8). RB1 CNV loss was observed in 10 out of 30 cSCLCs, with 80% (8/10) being SCLC. MYC CNV gain occurred in 3/8 AS-SCLC, 1/8 ADC/SCC, and 2/7 L-SCLC (Additional file 2: Table S6).

Analysis of significantly amplified or deleted regions was conducted at the chromosomal arm level (Additional file 2: Table S7). SCLC exhibited notable arm-level amplifications and deletions compared to paired ADC/SCC, involving chromosomes 19q, 18p, 20q, and 10q (all P < 0.05, Additional file 1: Figure S3A). Conversely, SCLC displayed heightened arm-level amplifications, including chromosomes 12p, 14q, 21p, and 21q (all P < 0.05), and a reduced degree of arm-level deletions compared to paired LCC, encompassing 3p, 5q, 10q, and 15q (all P < 0.05, Additional file 1: Figure S3B). Focal amplification of MYCL1 was identified in SCLC and paired LCC/LCNEC (Additional file 1: Figure S3C).

Numerous significantly focal CNVs around driver gene deletions in TP53, RB1, and RBL2 were evident in SCLC (Additional file 2: Table S8), along with deletions in CDKN2A and CDKN2B in paired ADC/SCC (Additional file 1: Figure S3D).

We conducted a comparative analysis of driver genes in cSCLC and their corresponding pure tumors using external data. Notably, no significant differences were observed in genomic profiles between pure tumors and matched tumor components of cSCLC. This similarity was particularly evident in key driver genes such as TP53, RB1, EGFR, and PTEN (Additional file 1: Figure S4A–C).

EGFR, a common driver gene in the Asian population enriched in ADC/SCC, showed clonal events in SCLC components of SCLC-ADC/SCC, indicating potential consanguinity or evolutionary correlation. Comparison of significant mutant driver genes between different components in cSCLC revealed enrichment of TP53 and RB1 in both SCLC and paired LCC/LCNEC. TP53 and EGFR were detected in both SCLC and paired ADC/SCC, while RB1 was exclusively enriched in SCLC (Additional file 1: Figure S4D, E), suggesting a pivotal role for RB1 in the formation of the SCLC component. These findings underscored the importance of TP53 and RB1 in cSCLC tumorigenesis.

Furthermore, we observed no statistical difference in TMB between tumor components in cSCLC and matched pure tumors (Additional file 1: Figure S4F). The hierarchical clustering tree based on the mutational spectrum across all cancer types (Additional file 1: Figure S4G) demonstrated that SCLC components of cSCLC-ADC/SCC exhibited genomic profile similarities with pure LUAD and LUSC. In contrast, LCC and L-SCLC displayed a closer relationship with pure SCLC, suggesting distinct evolutionary processes, with L-SCLC and AS-SCLC undergoing different evolutionary trajectories and the former being more closely related to pure SCLC.

To assess whether histological factors influenced the molecular classification of SCLC, we conducted detection based on the expression of ASCL1, NEUROD1, POU2F3, or YAP1. The ASCL1 subtype, a significant neuroendocrine regulator, comprised a substantial proportion of both AS-SCLC/L-SCLC and pure SCLC, with NEUROD1 and POU2F3 subtypes following in prevalence (Additional file 1: Figure S4H).

### Transcriptomic comparison in different components of cSCLC

Differentially expressed genes (DEGs) in SCLC versus paired ADC/SCC are illustrated in Fig. 2A. Significantly, numerous chemokine-related genes demonstrated downregulation in SCLC. These included CX3CL1, which activates the Src/FAK signaling pathway, thereby fostering the migration and invasion of lung cancer. Besides, downregulated genes such as CCL22 and CCR4 are chemokines derived from macrophages, while CXCL2 and CCR2 play roles in immunoregulatory and inflammatory processes. Additionally, downregulated genes in SCLC included MHC-II antigen presentation-related genes, such as CD74, CTSS, CIITA, HLA-DRA, HLA-E, and HLA-DPB1, crucial for CD4^+^ T cell-dependent immune responses. In contrast, EGFR was upregulated in ADC/SCC, while ASCL1 was upregulated in AS-SCLC. Additionally, in SCLC and paired LCC, five genes were upregulated, while 120 genes were downregulated (Fig. 2B).

Furthermore, Gene Set Enrichment Analysis (GSEA) revealed enrichment of MYC targets and E2F target-related pathways in AS-SCLC, while immune-related pathways were enriched in ADC/SCC (Fig. 2C). MYC targets and E2F targets-related pathways were also enriched in L-SCLC, while neuroactive ligand-receptor interaction and KRAS signaling pathways were enriched in LCC/LCNEC (Fig. 2D).

### TIME comparison in different components of cSCLC

**Fig. 3:**
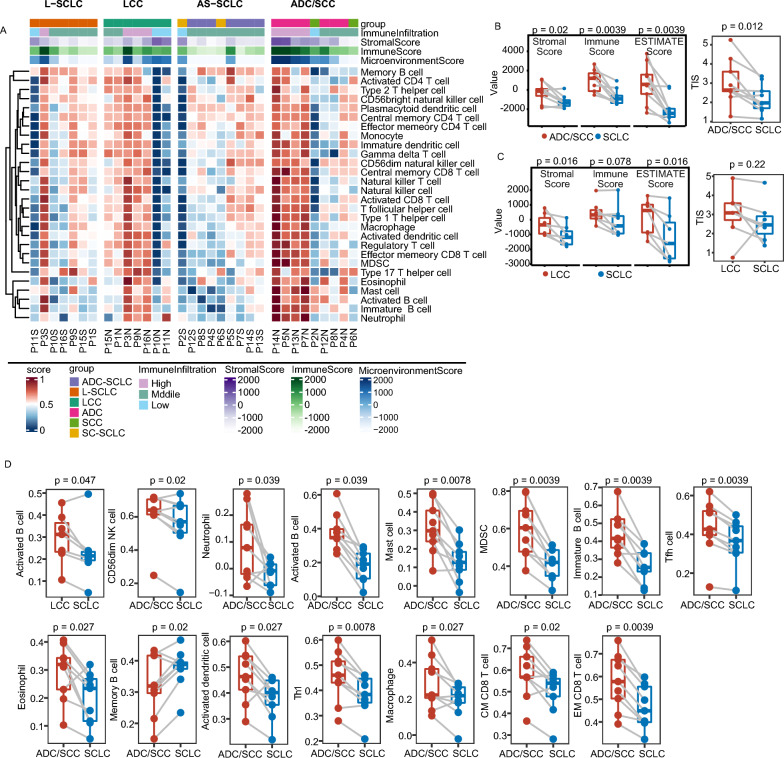
TIME Profile in cSCLC. **A** Heatmap of immune cell infiltration in cSCLC; **B** Comparison of stromal score, immune score, microenvironment score, and TIS score between SCLC and ADC/SCC; **C** Comparison of stromal score, immune score, microenvironment score, and TIS score between SCLC and LCC; **D** Boxplot showing significant differences in immune cell infiltration between two components of cSCLC

We applied a consensus clustering algorithm to categorize 32 samples into high, medium, and low levels of immune infiltrate based on immune cell infiltration. The majority (93.8%, 15/16) of SCLC were predominantly located in low or medium levels of immune infiltrate groups, whereas tumors with high levels of immune infiltration were primarily ADC/SCC (Additional file 2: Table S9, Fig. 3A). The stromal score, immune score, immune microenvironment score, and tumor inflammation signature score (TIS) of SCLC were all statistically lower compared with paired ADC/SCC (Fig. 3B), with the stromal score and tumor microenvironment score of LCC also higher than those of paired SCLC (Fig. 3C). A comparison of immune cell infiltration between SCLC and paired LCC/ADC/SCC is presented in Fig. 3D. Notably, immune-positive regulated immune cells in ADC/SCC exhibited significantly higher immune infiltration compared to paired SCLC, encompassing activated and immature B cells, macrophages, central memory (CM), and effector memory (EM) CD8^+^ T cells. In contrast, only activated B cells showed significantly higher immune infiltration in LCC/LCNEC compared to paired SCLC. In addition, infiltration level of immune cells, total cell infiltration score, stromal score, and TIS scores showed no statistical difference between L-SCLC and AS-SCLC (Additional file 1: Figure S5A–C).

Additionally, we selected 25 innate and adaptive immune pathways (Additional file 2: Table S10). All of these pathways were downregulated in SCLC compared with paired ADC/SCC, except for natural killer-mediated immunity. On the flip side, there was a statistically observed downregulation in chronic inflammatory response, the regulation of type 2 immune response, and Th1 immune response in SCLC as opposed to paired LCC (Additional file 1: Figure S5D). Furthermore, we compared the TIME between L-SCLC and AS-SCLC. L-SCLC exhibited a higher enrichment score of immune-related pathways (Additional file 1: Figure S5E) than AS-SCLC, including myeloid cell activation, JAK/STAT signaling, regulation of cytokine production, and inflammatory response. In summary, the results indicated that cSCLC and LCC tumors exhibited consistent TIME, while cSCLC and ADC/SCC tumors demonstrated TIME heterogeneity, with SCLC tending to be a "cold" tumor (less or no immune cell infiltration) and ADC/SCC tending to be a "hot" tumor (more immune cell infiltration).

### Clonal evolutionary origin of cSCLC

**Fig. 4:**
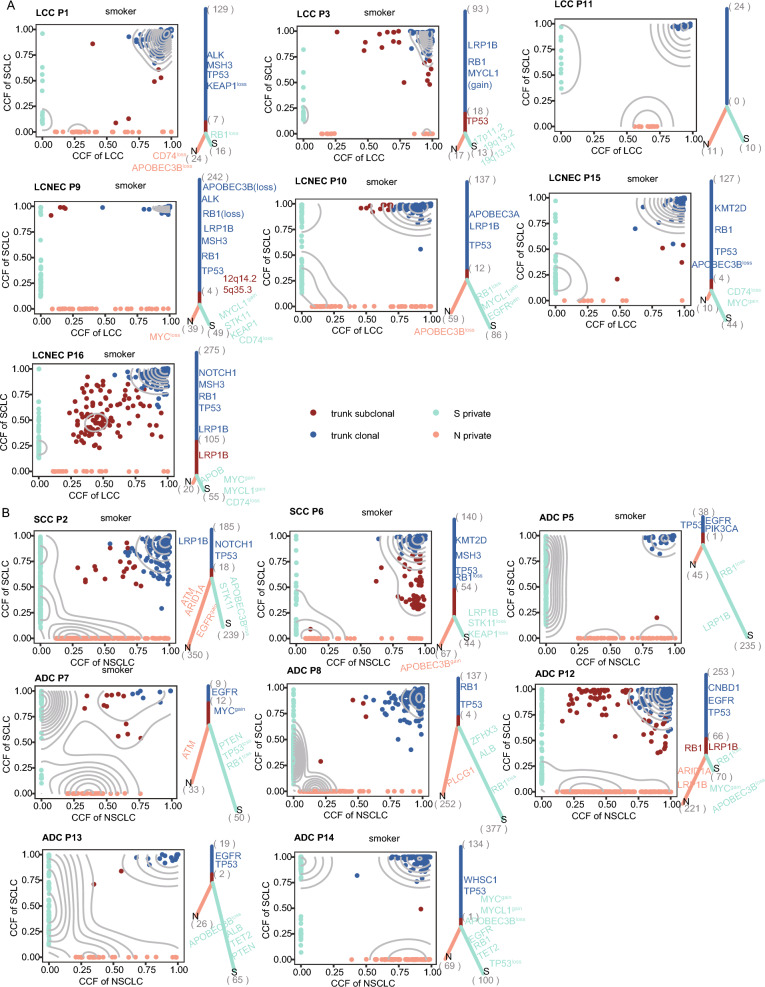
Phylogenetic Tree in cSCLC. **A** Density plot of mutations, CCF, and phylogenetic tree in L-SCLC; **B** Density plot of mutations, CCF, and phylogenetic tree in A/S-SCLC. Each point in the density plot on the left panel represents a mutation, with different colors indicating different positions from the phylogenetic tree in the right panel. The right panel displays the evolutionary tree of one patient, with the trunk clone, the trunk subclone, and the two branches indicated by different colors, respectively

The phylogenetic trees revealed that both histological components shared common mutations in all cSCLC tumors, suggesting a potential monoclonal origin for cSCLC (Fig. 4). Additionally, the trunk ratio was consistently higher in almost all cSCLC-LCC/LCNEC subtypes, ranging from 50 to 83%, compared to cSCLC-ADC/SCC subtypes, which ranged from 12 to 63% (Fig. 4A, B, Additional file 2: Table S11). This finding implied that the subclonal structure of SCLC was similar to that of paired LCC.

TP53 and RB1 mutations as trunk clonal events were nearly universal in all cSCLC-LCC/LCNEC tumors (Fig. 4A), occurring in the early stages of cSCLC-LCC/LCNEC tumor evolution (Additional file 1: Figure S6A, Additional file 2: Table S12). Consistent with a previous study (Fig. 4A) [27], LCNEC with TP53 and RB1 co-mutation exhibited a high level of immune cell infiltration, as observed in P3N, P9N, and P16N tumors. Tumors with TP53 and KEAP1 co-mutation (P1N) showed a moderate level of immune infiltration (Fig. 3A). Additionally, somatic mutations of LRP1B were detected in 57% (4/7) of the SCLC-LCC subtype and were identified as trunk clonal events.

In eight cSCLC-ADC/SCC tumors, EGFR mutations were predominantly identified as early-arising clonal events in the progression of cSCLC-ADC/SCC tumors (7 out of 11, Additional file 1: Figure S6B). TP53 mutations were clonal events in the majority (14 out of 16), with seven of them occurring early in the tumor evolution. Notably, LRP1B was primarily identified as a subclonal mutant gene in both SCLC and paired ADC/SCC, while it presented as a clonal mutant gene in SCLC-LCC tumors. TP53 and EGFR, as trunk clonal events, were identified in three SCLC-ADC tumors (P5, P12, and P13). Moreover, nearly all (4 out of 5) ADC/SCC tumors with EGFR mutation as clonal events exhibited a high level of immune cell infiltration (P5N, P7N, P13N, and P14N), while the matched SCLC tumors, with clonal EGFR mutations and RB1 inactivation, demonstrated low or moderate levels of immune cell infiltration (Fig. 3A).

LCC/LCNEC and L-SCLC exhibited a similar clonal structure with less selection pressure. Interestingly, ADC/SCC and AS-SCLC displayed a less similar clonal structure with higher selection pressure (Additional file 1: Figure S6C–F, Additional file 2: Table S13). Additionally, we calculated the dN/dS ratio on protein-coding regions to unveil the selection pressure on the two components in cSCLC tumors. The dN/dS ratio for both LCC and L-SCLC was less than 1, indicating negative selection and an indistinct transforming direction. The dN/dS ratio for AS-SCLC was 1.04, while the ratio for ADC/SCC was 0.96. Overall, SCLC components exhibited more evolutionary stability than ADC/SCC components in cSCLC-ADC/SCC tumors.

To explore whether tumor purity influenced the evolutionary tree structure, we conducted linear regression analysis and found no association between tumor purity and subclonal proportion (Additional file 1: Figure S6G). Similarly, there was no clear correlation between tumor cell proportion and subclonal proportion (Additional file 1: Figure S6H, I). Additionally, advanced tumors seemed to have higher subclonal proportions, although without statistical significance (Additional file 1: Figure S6J, K).

The proportion of lung epithelial cells in cSCLC revealed that ADC components mainly originated from AT2 cells, while SCC components mainly originated from basal cells. Pulmonary neuroendocrine cells (PNECs) accounted for the highest proportion in LCC, LCNEC, and SCLC (Additional file 1: Figure S7A). Considering driver genes, subclonal structure, lung epithelial cell proportions of different components in cSCLC, and tumor locations, we inferred that cSCLC might undergo two transformation modes.

The first mode involved a transformation from ADC to SCLC, as seen in P7, P12, and P14 (mode 1, Additional file 1: Figure S7B). The second mode involved a transformation from SCLC to SCC, as observed in P6 (mode 2, Additional file 1: Figure S7C). The cell of origin for these two transformation modes might differ. In mode 1, cSCLC might originate from PNECs, typically developing in central locations, while in mode 2, cSCLC might originate from AT2 and more commonly localize peripherally. cSCLC might represent an intermediate state in the process of SCLC transformation, potentially transforming into another histological tumor morphology through interactions with the TIME.

Notably, in P12, ADC and SCLC components shared TP53, RB1, and EGFR triple mutations and originated from a common ancestor clone. The cluster where the RB1 mutation was located was a subclonal event in ADC and a clonal event in SCLC. In drug-resistant EGFR-mutant LUAD, RB1 inactivation plays a critical role in SCLC transformation, suggesting that ADC might transform into SCLC under RB1 inactivation in P12 (Fig. 4B, Additional file 1: Figure S7D).

For P14, initially diagnosed as peripheral ADC (Additional file 1: Figure S7E) combined with SCC, the patient underwent right upper lung cancer radical resection. Recurrence occurred within a year, with staining confirming SCLC (Additional file 1: Figure S7F). Conversely, in P6, a cluster of subclonal events in SCLC corresponded to clonal events in paired SCC (Fig. 4B), involving Notch signaling-related genes (DTX3 and SNW1), Ras protein signal transduction (AKAP13, ALS2, and GPR55), neuron projection, and certain cellular components (SCN1A, SCN2A, and KIF1A). Abnormalities or dysfunctions in these pathways might be potential mechanisms for SCLC-to-SCC transformation. Additionally, a case report [28] has found a transformation from small cell to SCC in a thymic carcinoma patient, suggesting that although SCLC-to-SCC transformation is rare, it is possible to transform SCLC into SCC. Others could not infer the transformation mode (Additional file 1: Figure S7G).

### The heterogeneity of immune evasion capacity in cSCLC

**Fig. 5:**
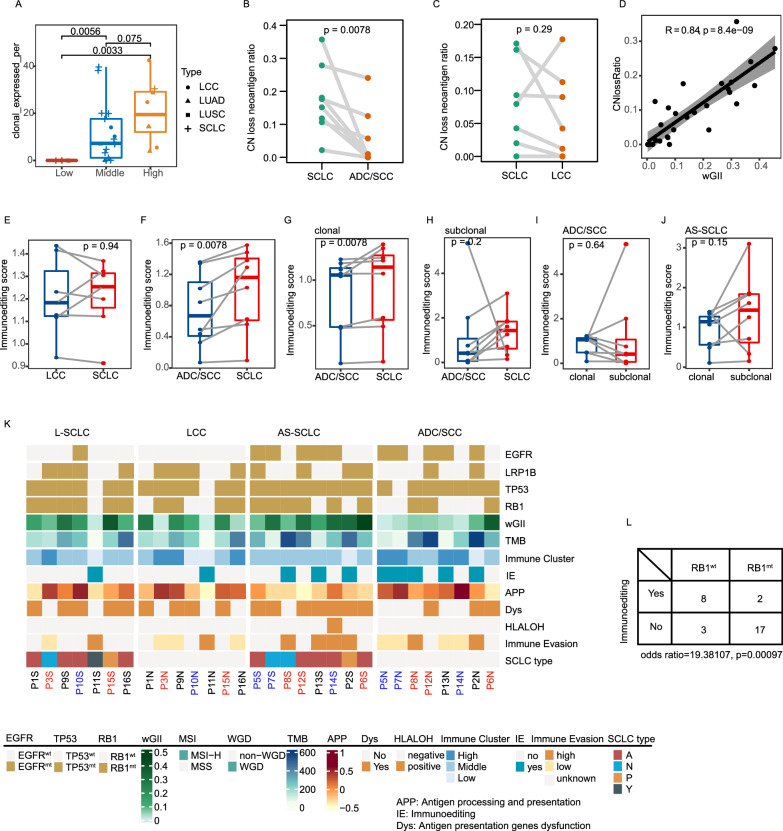
Mechanism of Immune Evasion in cSCLC. **A** Percentage of clonal expressed antigens among different levels of immune infiltration; **B** Copy-number loss ratios between LCC and paired SCLC; **C** Copy-number loss ratios between ADC/SCC and paired SCLC; **D** Correlation between copy-number loss ratios and wGII; **E** Immunoediting score between LCC and paired SCLC; **F** Immunoediting score between ADC/SCC and paired SCLC; **G** Immunoediting score of clonal mutations or **H** subclonal mutations between ADC/SCC and paired SCLC; **I** Immunoediting score between clonal mutations and subclonal mutations in ADC/SCC, or **J** AD-SCLC; **K** Overall overview of immune evasion in cSCLC; **L** Immunoediting score between patients with wild-type RB1 and those with mutant RB1

We delved deeper into the immune escape capacity in cSCLC and underscored that the primary immune escape mechanisms involved neoantigen depletion and/or defects in antigen presentation that disrupt tumor antigen recognition in tumors. Neoantigen depletion may arise from copy-number loss events and the suppression of transcriptions associated with neoantigen production. We calculated the odds ratio of the occurrence of an expressed neoantigen and assessed neoantigen depletion in cSCLC in our study. At the cohort level, ADC/SCC, AS-SCLC, and LCC exhibited significant expression of neoantigens compared to L-SCLC (Additional file 1: Figure S8A, P < 0.001, P < 0.001, and P = 0.03, respectively). Moreover, when tumors were divided by levels of immune cell infiltration, those with high or intermediate levels of immune infiltration were more likely to generate neoantigens (Additional file 1: Figure S8B, both P < 0.001). Neoantigens were less likely to occur in genes consistently expressed across all samples of L-SCLC, ADC/SCC, and AS-SCLC (Additional file 1: Figure S8C, P = 0.02, P < 0.001, and P = 0.01, respectively). However, neoantigens of tumors with high and middle levels of immune infiltration were less likely to be expressed in consistently expressed genes (Additional file 1: Figure S8D, P = 0.03 and P < 0.001, respectively), and neoantigen mutations in genes consistently expressed in this study were most reduced among tumors with middle and low levels of immune infiltration. Conceivably, tumors with high immune infiltration levels exhibited more clonally expressed neoantigens than those with middle or low levels of immune infiltration (Fig. 5A, High versus Middle, P = 0.075; High versus Low, P = 0.003; Middle versus Low, P = 0.0056). This was consistent with the above findings, indicating that clonally expressed neoantigens were the main contributors to an active TIME.

We also utilized Fisher’s test to calculate the likelihood of copy-number neoantigen depletion. There was no significant difference at the tumor level (Additional file 1: Figure S8E), the middle-level immune infiltration group was more likely to experience copy-number loss of neoantigens (Additional file 1: Figure S8F). L-SCLC and middle-level immune infiltration groups were more likely to undergo clonal copy-number loss of neoantigens (Additional file 1: Figure S8G, H). No statistical difference was observed for likely to experience subclonal copy-number loss of neoantigens either at tumor-type level or at different immune infiltration level (Additional file 1: Figure S8I, J). Furthermore, the percentage of neoantigens occurring CNV in ADC/SCC was lower than in paired SCLC (P = 0.0078, Fig. 5B), while no statistical difference was observed between SCLC and LCC (P = 0.29, Fig. 5C). Consistently, the wGII and the number of neoantigens undergoing CNV loss events exhibited a favorable linear correlation (Fig. 5D, R = 0.84, P = 8.4e-09), suggesting that neoantigen depletion in SCLC was strongly associated with chromosomal instability (CIN).

Immunoediting, reported as a neoantigen-directed mechanism for tumor immune escape, is assessed through immunoediting scores representing the overall capacity of HLA alleles to edit mutations. This considers the antigens they can bind and the level of editing exhibited for a subset of antigens. Using a published method to quantify DNA immunoediting in each tumor sample, we observed no significant difference in the immunoediting score between SCLC and paired LCC (P = 0.94, Fig. 5E). However, the immunoediting score of SCLC was significantly higher than that of paired ADC/SCC (P = 0.0078, Fig. 5F), possibly influenced by neoantigens generated through clonal mutations (P = 0.0078, Fig. 5G) rather than subclonal mutations (Fig. 5H). In addition, the immunoediting score between clonal and subclonal mutations showed no significant difference, either in ADC/SCC or the paired SCLC (Fig. 5I, J). The phenomenon of immunoediting and disruptions to antigen presentation, whether through HLA LOH or variations affecting the stability of the major histocompatibility complex (MHC) and the HLA enhanceosome, can contribute to immune evasion. The assessment of immune evasion capacity involved a comprehensive evaluation of immune cell infiltration, as well as mechanisms related to immune escape, such as disruptions in neoantigen presentation and an immunoediting score. Overall, AS-SCLC tumors exhibited a notably high level of immune evasion, as depicted in Fig. 5K (L-SCLC: 1, LCC: 1, AS-SCLC: 4, ADC/SCC: 2).

### RB1 might be the main factor contributing to tumor heterogeneity in cSCLC

**Fig. 6:**
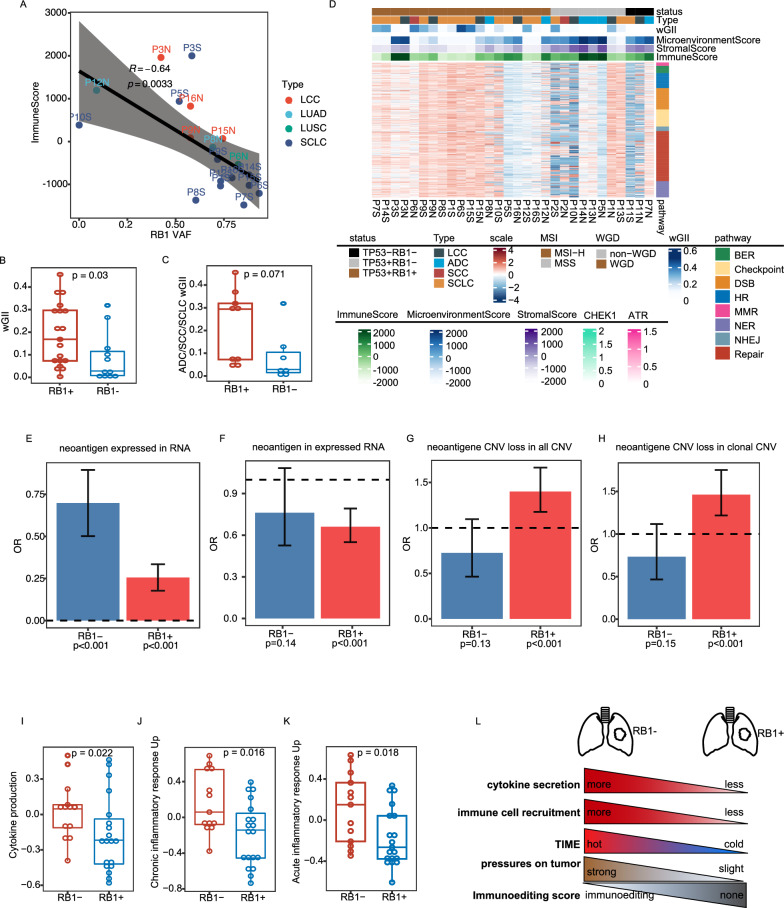
RB1 Mechanism in cSCLC. **A** Association between RB1 VAF and immune score; **B** wGII between wild-type RB1 and mutant-type RB1 in the whole cohort; **C** wGII in SCLC-ADC/SCLC subtypes; **D** Heatmap of DDR gene expression; **E** Likelihood of occurring neoantigen expressed in tumors with wild-type RB1 and mutant-type RB1; **F** Likelihood of generating neoantigen in consistently expressed genes in tumors with wild-type RB1 and mutant-type RB1; **G** Likelihood of neoantigen occurring in all copy number loss regions in tumors with wild-type RB1 and mutant-type RB1; **H** Likelihood of neoantigen occurring in clonal copy number loss regions in tumors with wild-type RB1 and mutant-type RB1; **I** Likelihood of neoantigen occurring in subclonal copy number loss regions in tumors with wild-type RB1 and mutant-type RB1; **J** Enrichment score of cytokine production; **K** Chronic inflammatory response up; **L** Acute inflammatory response up pathways between wild-type RB1 and mutant-type RB1; **M** Influence of RB1 alteration status on TIME

Based on the previously discussed genomic profile and TIME of SCLC, it exhibited a genomic profile and TIME similar to paired LCC, whereas there was a notable divergence in genomic features compared to paired ADC/SCC. Examining the relationship between pairwise immune and somatic mutation distances revealed no significant correlation (R = 0.2, P = 0.47, Additional file 1: Figure S9A). Consistent with findings by Knudsen [29] and as elucidated above, RB1 emerged as a recurrent genomic variation in cSCLC. Interestingly, the variant allele fraction (VAF) of RB1 was negatively associated with immune cell infiltration (Fig. 6A, P = 0.0033, R = − 0.64). These findings suggested that RB1 inactivation could potentially serve as a biomarker influencing the heterogeneity of the TIME in cSCLC. RB1 inactivation induces CIN by upregulating MAD2, leading to aneuploidy [30, 31]. Notably, RB1 inactivation appeared to be a key factor contributing to CIN in SCLC, supported by the observation that patients harboring RB1 inactivation exhibited higher wGII scores than those with wild-type RB1 (P = 0.03, Fig. 6B). Furthermore, there was no significant difference in wGII between patients with RB1 inactivation and those with wild-type RB1 when considering concurrent TP53 inactivation (P = 0.22, Additional file 1: Figure S9B), underscoring the pivotal role of RB1 over TP53 in CIN.

When exclusively analyzing SCLC-ADC/SCC tumors, wGII scores showed marginal statistical significance (P = 0.071, Fig. 6C). This trend was consistent in patients with co-occurring TP53 inactivation (P = 0.14, Additional file 1: Figure S9C). Interestingly, RB1 inactivation in tumors, regardless of TP53 status, did not correlate with WGD (Additional file 1: Figure S9D–G).

Recent studies have underscored the therapeutic potential of targeting the DNA damage response (DDR) pathway in SCLC as a promising strategy [32]. Notably, genomic variations in the DDR pathway did not exhibit significance when comparing LCC to L-SCLC and ADC/SCC to AS-SCLC (Additional file 1: Figure S9H).

An intriguing observation was the upregulation of several DDR-related genes in AS-SCLC tumors, including SSRP1, SMC1A, RAD21, PARP1, EXO1, CLSPN, BRIP1, and LMNB1. Among these, the role of PARP1 in DNA break repair has gained attention as a promising therapeutic target [33]. In ADC/SCC, HMGA2, a gene crucial in proliferation, metastasis, and epithelial-mesenchymal transition (EMT) in lung cancer, exhibited upregulation (Additional file 1: Figure S9I) [34].

In alignment with a recent study, TP53 and RB1 inactivation were associated with aberrant expression of DDR genes in SCLC [35]. Our results revealed that the gene expression of all DDR pathways in TP53^+^RB1^+^ patients was higher compared to those with TP53^+^RB1^−^ and TP53^−^RB1^−^, concomitant with elevated wGII and WGD, and lower TIME scores (Fig. 6D, Additional file 1: Figure S9J).

Notably, tumors with wild-type RB1 exhibited a higher likelihood of being active in immunoediting compared to those with mutant RB1 (odds ratio: 19.38, P = 0.00097, Fig. 5L). Moreover, tumors with mutant RB1 demonstrated a higher propensity for neoantigen depletion through CNV loss events and suppression of transcript expression, in contrast to tumors with wild-type RB1 (Fig. 6E–H).

RB1 status appears to influence the TIME, including chemokine/cytokine secretion and immune cell dynamics [36]. Our findings revealed that patients with RB1 inactivation exhibited lower levels of cytokine production (P = 0.022, Fig. 6I), chronic inflammatory response (P = 0.016, Fig. 6J), and acute inflammatory response (P = 0.018, Fig. 6K) than patients with wild-type RB1. RB1 inactivation was associated with reduced cytokine secretion, leading to diminished recruitment of immune cells and the gradual establishment of a 'cold' TIME (characterized by reduced or no immune cell infiltration). Consequently, the TIME pressure on the tumor diminished over time, making tumors less prone to generating immunoediting (Fig. 6L). This observation also provided insight into why SCLC tumors demonstrated greater activity in immunoediting compared to their paired ADC/SCC counterparts.

## Discussion

In the present study, we employed a combination of omic strategies to thoroughly investigate the genomic origin and TIME of cSCLC. The genomic profiling of cSCLC has been explored in several studies, incorporating diverse component analyses within the entire tumor structure [6–8, 37, 38]. However, to the best of our knowledge, none of these studies have assessed the heterogeneity among different tumor components in cSCLC. Therefore, a more comprehensive analysis of cSCLC is imperative. The current study strongly suggested that cSCLC exhibited intratumoral heterogeneity when comparing genomic alterations, TIME, and immune evasion capacity.

In this investigation, we enrolled 16 patients with cSCLC and conducted genomic and transcriptomic analyses of 32 samples obtained through laser microdissection of two distinct components in cSCLC. Throughout our comprehensive analysis, several intriguing features came to light. We observed that cSCLC harbored significant genomic alterations and oncogenic mutations, which varied across LCC, LCNEC, SCLC, and ADC/SCC, featuring TP53 and RB1 mutations in SCLC and LCC/LCNEC, MYC CNV gain in SCLC, and EGFR mutations in ADC/SCC or paired SCLC. Nonetheless, the genomic features of tumor components in cSCLC were akin to the matched pure tumors. While EGFR mutations were infrequent in pure SCLC, our results were surprising in revealing that EGFR mutations were more likely to occur frequently and act as early clonal drivers in SCLC combined with ADC subtypes, in contrast to pure SCLC.

Although the genomic landscape demonstrated robust consistency in SCLC components and LCC components of cSCLC-LCC, both exhibiting neuroendocrine properties, LCC components of SCLC-LCC subtypes displayed two genomic subgroups with specific transcriptional patterns, defined as TP53 and RB1 co-mutation group and TP53 and KEAP1 group.

The interaction between the tumor and the TIME plays a pivotal role in predicting clinical outcomes in lung cancer [39, 40], with immune-evasion capacity emerging as a prognostic biomarker [41, 42]. Despite its significance, there is currently no comprehensive literature detailing the immune evasion capability of cSCLC. Therefore, we employed a variety of methods to assess the immune capacity of cSCLC, including antigen preprocessing and presentation, as well as immune cell infiltration. Our findings illuminated that cSCLC exhibited heterogeneity in the intra-TIME, potentially influenced by copy-number loss of neoantigens and variations in RB1. This study represented a novel exploration into the immune dynamics of cSCLC, shedding light on their unique characteristics in the context of immune response.

Firstly, SCLC components of SCLC-ADC/SCC tumors demonstrated a higher immune evasion capacity than paired ADC/SCC components. Furthermore, SCLC-ADC/SCC exhibited more significant disparities in immune evasion capacity (displaying a high level of immune evasion capacity) compared to SCLC-LCC. Immunoediting, a neoantigen-directed mechanism of tumor immune escape, has been reported as well [43]. Immunoediting scores represent the overall ability of HLA alleles to edit mutations, considering the repertoire of antigens they can bind and the level of editing they exhibit for the subset of antigens [44]. We employed a previously established methodology to quantify the degree of DNA immunoediting in each tumor sample [45]. Moreover, we found no significant difference between the immunoediting score in SCLC and paired LCC (P = 0.94). However, the immunoediting score of SCLC was significantly higher than that of paired ADC/SCC (P = 0.0078), possibly dominated by clonal mutations associated with generated neoantigens.

Despite the observed heterogeneity in genomic alterations and TIME among different tumor components of cSCLC, we posited that these components might share a common clonal origin due to the high frequency of shared mutations. Our results strongly indicated that cSCLC originated from a monoclonal source, suggesting that a single pluripotent clone underwent differentiation into different components following the acquisition of one or more key mutations. Unsupervised clustering of mutational spectra further supported a close relationship between SCLC-ADC/SCC and ADC/SCC, raising the possibility that SCLC and ADC/SCC components stem from the same progenitor.

Furthermore, considering four dimensions of driver genes, subclonal structure, the proportion of lung epithelial cells, and tumor location, we proposed that cSCLC might undergo two transformation modes. Mode 1 involved a transformation from ADC to SCLC, originating from AT2 and typically being more peripherally localized. On the other hand, mode 2 entailed a transformation from SCLC to SCC, potentially originating from PNECs and usually developing in a central location. Our findings illuminated that cSCLC represented an intermediate state in the process of SCLC transformation and had the potential to undergo a complete transformation into another histological tumor morphology through interactions between the tumor and the TIME surrounding it.

Furthermore, our investigation suggested that RB1 might be a key factor driving the transdifferentiation process. This hypothesis gained support from the phylogenetic tree of P12, a triple mutant ADC (EGFR/RB1/TP53), which exhibited sub-clone formation following the acquisition of RB1 deletion. Triple mutant ADC is recognized as being at a higher risk of transforming into SCLC [46], making it a compelling piece of evidence for the role of RB1 loss in the formation of different components in cSCLC. This notion aligned with a recent study that highlights the involvement of RB1 and TP53 in driving AT2 cells to SCLC [47].

Our study also revealed that RB1 was implicated in immune cell infiltration and immune functions. Results indicated that ADC/SCC with TP53 and RB1 co-mutation exhibited low or moderate levels of immune cell infiltration. Interestingly, we observed a negative correlation between the variant allele frequency (VAF) of RB1 alterations and levels of immune cell infiltration, with SCLC tumors exhibiting higher VAF of RB1 mutations compared to LCC with mutant-RB1. ADC/SCC with high levels of immune cell infiltration commonly features EGFR mutations as clonal events and wild-type RB1, suggesting that RB1 and EGFR may play opposing roles in the TIME. Moreover, RB1 mutations were associated with the downregulation of chemokine, antigen-presenting-related genes, and innate immune pathways. Tumors with mutant-type RB1 exhibited neoantigen depletion through copy number loss events and transcriptional suppression, while tumors with wild-type RB1 demonstrated greater activity in immunoediting. These findings aligned with a previous report suggesting that RB1 loss may lead to the downregulation of a significant subset of immune genes, including those encoding immune cell surface receptors, complement components, and cytokines [48].

Considering the high wGII of SCLC-ADC/SCC with RB1 mutation, we hypothesized that the high CIN resulted in RB1 deletion, subsequently leading to transdifferentiation and an intra-heterogeneous TIME. Additionally, DNA repairing and strand exchange in the homologous recombination (HR) pathway may affect the expression of immune checkpoint molecules after DNA damage, leading to immunosuppression [35].

It is essential to acknowledge the limitation of our study, primarily stemming from the relatively small sample size due to the rarity of cSCLC. This limitation may introduce bias and potentially impact the generalizability of our results. In our exploration of the relationship between genomic features and the TIME, we calculated a distance measure in both genomic and immune space for all pairwise combinations of tumor regions from the same tumor. However, we found no statistical difference, which could be attributed to the limited sample size, particularly in detecting linked changes in immune-related genes and immune infiltration. As a result, our findings should be interpreted with caution and require validation in a larger-scale cohort to ensure robustness. Additionally, it's important to note that the molecular heterogeneity of tumors is dynamic and continuously evolving under external pressures. While a dynamic biopsy for longitudinal monitoring is crucial, the high cost and limited applicability of such an approach led us to analyze specimens obtained at the time of diagnosis. Despite this constraint, our use of combinational omic strategies provided a comprehensive and vivid description of the genomic profile, origin, and TIME.

In conclusion, this study represented a preliminary exploration of the genomic origin of different components in cSCLC through integrated analysis, offering novel insights into the evolutionary process of cSCLC. Our data suggested that cSCLC had a monoclonal origin, and RB1 played a crucial role in driving transdifferentiation from the ADC/SCC to the SCLC component, influencing immune evasion. However, these findings warrant further validation in larger cohorts to enhance the robustness and reliability of the results.

### Supplementary Information

**Additional file 1.**. **Table S1.** Clinical details of cSCLC cases. **Table S2.** All somatic mutations of cSCLC samples. **Table S3.** Potential driver genes in cSCLC samples. **Table S4.** Other recurrently mutant genes in cSCLC samples.** Table S5.** Predicted neoantigens in cSCLC samples. **Table S6.** Somatic copy number variations in cSCLC samples. **Table S7.** Significant broad copy number variations in cSCLC samples. **Table S8.** Significant focal copy number variations in cSCLC samples. **Table S9.** CCF and clone clusters of somatic non-synonymous mutations in cSCLC samples. **Table S10.** clonality of somatic non-synonymous mutations in cSCLC samples. **Table S11.** Somatic evolutionary timings of mutations. **Table S12.** Immune cell fraction of cSCLC samples. **Table S13.** Immune-related pathways enrichment score of cSCLC samples.

**Additional file 2.** **Figure S1.** An overview of the design of this study. A) Schematic diagram of study design; B) Schematic diagram of laser microdissection, left picture represents the IHC staining of cSCLCs before laser microdissection, the right represents the IHC staining of cSCLCs after laser microdissection, the yellow dotted line represents the lung adenocarcinoma component to be separated. **Figure S2.** Genomic biomakers in cSCLCs. A) Distribution of the 96 mutation types combined across cSCLCs; Mutational signatures B) between LCC and paired SCLC components; C) between ADC/SCC and paired SCLC components; D) between different tumor types; E) between L-SCLC and AS-SCLC; Boxplot in TMB F) and TNB G) between L-SCLC and AS-SCLC; Boxplot in TMB H) and TNB I) at the tumor types level; wGII comparisions J) at the tumor types level, and K) between L-SCLC and AS-SCLC. The percentage of occurred whole genome doubling L) between LCC and paired SCLC components; M) between ADC/SCC and paired SCLC components. **Figure S3.** Copy number variations profile of cSCLCs. Frequencies of copy number variations in chromosome arm-level of A) SCLC and paired ADC/SCC, B) SCLC and paired LCC. Recurrent focal CNVs in C) SCLC and paired LCC, D) SCLC and paired ADC/SCC, with potential CNV drivers annotated on the circos plot. Focal amplications were marked by red color, Focal deletions were marked by blue color. **Figure S4.** Driver genes between pure tumors and cSCLCs. A) The MutSigCV p-value of drivers A) between LCC components and pure LCC tumors; B) between ADC/SCC components and pure LUAD/LUSC tumors; C) between SCLC components and pure SCLC tumors; D) between LCC components and paried SCLC components; E) between ADC/SCC components and paired SCLC tumors. F) The tumor mutation burden between pure tumors and cSCLC. The frequency of G) TP53, H) RB1, I) EGFR between pure tumors and cSCLC. J) Hierarchical clustering based on mutational spectrum on cSCLC and the corresponding pure tumors. K) SCLC subtypes in SCLC components and pure SCLC tumors. **Figure S5.** Enrichment of Immune-related pathways and immune cell infiltration in CSCLCs. The comparisions of A) immune cell infiltration, B) tumor immune microenvironment, and C) TIS between L-SCLC and AS-SCLC components; D) The enrichment score heatmap of immune-related pathways in CSCLCs, and statistics p-value between different components; E) The comparisions of enrichment scores of immune-related pathways between L-SCLC and AS-SCLC components. **Figure S6.** Clonality in CSCLCs. The bar plots showed clonal types of each driver genes in different components of A) SCLC-LCC subtypes and B) SCLC-ADC/SCC subtypes; C) The distribution of somatic mutations clonality between different components in each cSCLC tumor; The comparisions of D) no selection, E) positive selection in non-SCLC components, and F) positive selection in SCLC components between SCLC-LCC subtypes and SCLC-ADC/SCC subtypes; The assocations between G) tumor purity, H) LCC/SCC tumor proporition, I) ADC/SCC/SCLC tumor proporition, J) tumor stages of non-SCLC components, K) tumor stages of SCLC components and the ccorresponding subclonal ratios. **Figure S7.** Epithelial cell components of cSCLCs and tranformation modes in CSCLCs. A) the proportion of epithelial cell components in cSCLCs; B) subclonal structure of patients which are transformed from ADCs to SCLCs; C) subclonal structure of patients which are transformed from SCLCs to SCCs; D) The evolutionary fishplot of P12 patient; E) 3D reconstruction of P14's CT scan; F) IHC staining of primary tumor and recurrent tumor of P14. G) subclonal structure of patients which cannot be inferred the transformation direction. **Figure S8.** Neoantigen depletion in CSCLCs. The odds ratio to occur neoantigen expressed in A) different tumor subtypes and B) different levels immune cell infiltration. The odds ratio to generate neoantigen in consistently expressed genes C) different tumor subtypes and D) different levels immune cell infiltration. The odds ratio to neoantigen occurred in all copy number loss region in E) different tumor subtypes and F) different levels immune cell infiltration. The odds ratio to neoantigen occurred in clonal copy number loss region in G) different tumor subtypes and H) different levels immune cell infiltration. The likely to neoantigen occurred in subclonal copy number loss region in I) different tumor subtypes and J) different levels immune cell infiltration. **Figure S9.** The associates between RB1 and genomic biomarkers in cSCLCs. A) The correlation between immune distance and the pairwise genomic distance. The weighted chromosome instability comparisons between different TP53 and RB1 alterations status B) in the whole cohort and C) SCLC-ADC/SCC cohort. Comparisons of the whole genome doubling between different TP53 and RB1 alterations status D) in the whole cohort and E) SCLC-ADC/SCC cohort. H) DDR pathways alterations between SCLC components and LCC/ADC/SCC components. I) Differently expressed genes of DDR pathways in SCLC versus ADC/SCC. J) The enrichment score of DDR pathways among tumors with different alteration status of TP53 and RB1.

## Data Availability

The datasets generated during and/or analysed during the current study are available from the corresponding author on reasonable request. The human WES and RNA-seq data generated during this study are accessible at the National Genomics Data Center, with the accession numbers provided under HRA003679.
