# Supplementary material for: Genomic and transcriptomic profiling of combined small-cell lung cancer through microdissection: unveiling the transformational pathway of mixed subtype
Source: J Transl Med. 2024 Feb 21;22:189. doi: 10.1186/s12967-024-04968-4 (PMC10880258; doi:10.1186/s12967-024-04968-4)

**Supplementary Information (SI)**

**Genomic and** **Transcriptomic Profiling of Combined Small Cell Lung Cancer Reveals** **different evolution process of different subtypes**

Wenjuan Ma, Ting Zhou, Mengmeng Song, Jiaqing Liu, Gang Chen, Jianhua Zhan, Liyan Ji, Fan Luo, Xuan Gao, Pansong Li, Xuefeng Xia, Yan Huang, Li Zhang

**Supplementary Methods:**

***Tumor tissue microdissection***

The morphological patterns that indicate adenocarcinoma (ADC) differentiation include lepidic, acinar, papillary, micropapillary, solid, enteric pattern as well as invasive mucinous adenocarcinoma and colloid adenocarcinoma. Squamous cell differentiation can be demonstrated morphologically by presence of keratinization, squamous pearls, and intercellular bridges. In poorly differentiation carcinoma with the solid pattern where IHC biomarkers including TTF1 and NapsinA stains are positive but P40 is negative, the diagnosis is identified as ACC. If the tumor is P40 positive but TTF1 negative, the diagnosis should favor SCC. Morphological features of neuroendocrine carcinoma consist of peripheral palisading, organoid nesting pattern or rosette-like structure. The discrimination of small cell lung carcinoma (SCLC) from large cell neuroendocrine carcinoma (LCNEC) is based predominantly on light-microscopic features, with LCNEC generally having more abundant cytoplasm, polygonal cell shape and distinct nucleoli, meanwhile, expression of neuroendocrine marker is required for the diagnosis of LCNEC, and the expression of at least one neuroendocrine marker such as synaptophysin (SYN), chromogranin A (CGA) and CD56 is typically diffuse. Classically, SCLC is considered a light-microscopic diagnosis whose features consists of scant cytoplasm, poorly defined cell borders, and finely granular nuclear chromatin, and immunostains are not required to confirm the diagnosis. However, most SCLCs will stain for neuroendocrine markers including SYN, CGA and CD56, with SCLC usually expressing at least one of these markers.

Manual microdissection was carried out in the area where the ADC, SCC, LCNEC and SCLC are clearly separated. Laser-capture microdissection was carried out on selective samples that contained both histologic components that are not separable by normal manual microdissection.

***Whole-exome sequencing***

For each region of the patient, DNA was extracted from the FFPE kit (Promega) according to the manufacturer's instructions. We constructed the sequencing libraries from native DNA using the xGen^®^ Exome Research Panel (Integrated DNA Technologies, Iowa, IA, USA) and the NEB Next Ultra DNA Library Prep Kit (Lot: NEB-0311611, NEB, UK) with a KAPA polymerase (KapaBiosystems, Wilmington, MA, USA). Whole-exome sequencing was performed using GeneSeq-2000 (Geneplus-Suzhou, Suzhou, China), with 100-bp paired-end reads. The peripheral blood monocyte cell DNA served as a control to exclude germline mutations.

Fastp was used to filter out low quality reads, too short reads, and cut off adapters from raw reads to obtain clean reads, clean reads were aligned to human reference genome (hg19) using Sentieon-genomics pipeline (version sentieon-genomics-201808). Read mapping was performed by bwa mem. LocusCollector and Dedup algorithms were used to mark and remove duplicate reads. sQualCal was used in the base quality score recalibration stage. Realigner performed local realignment around indels on the BAM file after alignment, sorting and deduping. DNA scope was used to call germline SNV and InDel after only alignment and deduplication. ApplyVarCal was used in the Variant Quality Score Recalibration (VQSR) stage.

***RNA next generation sequencing (RNA-seq)***

NEBNext rRNA Depletion Kit (Human/Mouse/Rat) (NEB #Z1955E) was chosen to remove the targeted ribosomal RNA (rRNA). All RNA with a percentage of RNA fragments > 200 nucleotides (DV200) ≤ 50% skipped fragmentation and proceeded to library preparation. After rRNA depletion and fragmentation, cDNA synthesis and NGS library preparation were performed using NEBNext^®^ Ultra™ II Directional RNA Library Prep Kit (NEB#E7760L). The library was quantitated using Qubit 3.0 (life Invitrogen, USA) and quality was assessed with LabChip GX Touch (PerkinElmer, USA). rRNA was discarded by aligning clean reads to the NCBI rRNA database by using bowtie2 (version: 2.2.8) after removing terminal adaptor sequences and low-quality data by using fastp (version: 0.20.0). Clean reads without rRNA were aligned to the reference human genome (hg19) through STAR (version 2.6.1d). StringTie (version 2.0.6) was used for gene expression quantification and gene expression level was normalized by TPM (transcripts per million).

***Somatic mutation calling***

Somatic Single nucleotide variants (SNVs) and small insertions and deletions (InDels) were called by GATK Mutect2 (version 4.1.4.1) software. A mutation was filtered out, if any of the following conditions was met: (I) a mutation site was not a predefined hot site and not in predefined hot regions which contained EGFR exon 19/20, ERBB2 exon 20, and MET exon 14, and variant allele frequency (VAF) of the mutation less than 0.03. (II) a mutation occurred frequently in most human cancer, but it was not real mutation that caused cancer or occurred frequently in normal tissues, and VAF was less than 0.05. (III) a mutation was present in >1% of the population in the 1000G (1000 Genomes Project, version phase 3), 1000G-EAS (1000G East Asian Project), ExAC database (The Exome Aggregation Consortium, version 0.2), or ExAC-EAS (ExAC East Asian). (IV) a mutation was in the predefined blacklist database. Microsatellite instability (MSI) was calculated using a published MSI sensor tool (v0.2).

***Copy number variation***

Somatic copy number variation (CNV) was identified with FACETS (v0.5.11). CNV gains were defined as segments with copy number/ploidy > log2(2.8/2), while CNV losses were segments with copy number/ploidy < log2(1.4/2). To calculate the CCF of each copy number event, simply divide the cf.em column by the purity of the sample. In some cases, the cf.em value may be slightly higher than the purity value due to noise, which in that case the CCF estimates could be rounded to 1.0 (100%). A CCF greater than 1.0 was set to 1.0. CCF greater than 0.7 was considered clonal CNV, otherwise it was a subclonal CNV. GISTIC analysis was performed to identify significant arm-level and focal-level CNV. copynumber (1.32.0) R package is used for visualization of the copy number segments.

***Tumor purity, ploidy, and WGD***

The tumor purity, ploidy and whole-genome doubling (WGD) for each sample were estimated by ABSOLUTE (v1.2).

***Weighted Genomic Instability Index***

Chromosome instability (CIN) could be measured by weight genomic instability index (wGII) [22] based on tumor ploidy, chromosome length, and copy number variation which implemented by CINcalc. The calculation formula for wGII was as follows:

$$wGII= \frac{\sum_{c=1}^{m} \sum_{i}^{k} \frac{l_{i}}{L_{c}}}{m}$$

where p was tumor ploidy, m was the number of chromosomes, k was the number of the CNV segments for each chromosome, L_c_ was chromosome length, and l_i_ was the length of the CNV segment. We set amplified segment threshold as 0.49 (log2(1.4)), loss segment threshold as -0.51 (log2(0.7)).

***Mutational signatures***

We used sigminer R package to extract mutational signatures based on COSMIC signature V3 (https://cancer.sanger.ac.uk/signatures/). Specifically, First, we used *read_maf* method to load somatic mutations and tallied components in each sample, and generated a sample-by-component matrix by using *sig_tally* method. Then, we used *sig_auto_extract* method to automatically obtain optimal signature number and extract signatures based on bayesian NMF. After extracting signatures, *get_sig_similarity* was performed to compare the identified signatures with COSMIC signatures (version 3) to obtain their etiologies. We also could use *sig_fit* method to compute absolute and relative exposure of each identified signature for each sample.

***Phylogenetic tree construction***

The phylogenetic tree of each patient was constructed based on all non-synonymous somatic mutations in all samples of the patient, with selected potential driver genes marked in the trunk and branch, the length of trunk and branches representing the numbers of mutations. Trunk mutations occurred in all samples of the patient, branch mutations only occurred in one sample, the length of each tree's branch was calculated according to the number of mutations on each branch. PhylogicNDT (Phylogic N-Dimensional with Timing) Clustering method [23] was used to calculate SNV CCF and identify clusters of mutations with consistent cancer cell fractions across two samples from one patient and determine the cancer cell fraction (CCF) posteriors for each cluster based on tumor purity and copy number segment. The cluster with the maximum CCF was the clonal cluster, in which all mutations were clonal mutations, and the other clusters were subclonal clusters.

Trunk events are mutations present in all the samples of each patient. According to CCF, trunk events are classified into trunk clonal and trunk subclonal events in further.

***Construction of subclonal structure***

After determining the cancer cell fraction (CCF) for each cluster through the PhylogicNDT Clustering step, and then removed clusters with less than 5 mutations, finally, buildTree method used the clusters and mutations CCF results to assemble likely tree structures by moving individual tree branches (subclones) within each iteration according to a multinomial probability (based on the pigeonhole rule) of the tree branch (subclone) being integrated into a specific position within the tree. Clonal abundance of each sample was also generated by the buildTree step.

***Evolutionary selection pressure***

We calculated the number of non-selective mutations, positive selective mutations in SCLC component, positive selective mutations in ADC/SCC/LCC component based on clonality and CCF of mutations for each patient. "clonal both" or "subclonal both" was defined as either clonal or subclonal mutations in both components for each patient. "novel clonal in LCC/ADC/SCC" or "novel subclonal in LCC/ADC/SCC" was defined as mutations were clonal or subclonal in LCC/ADC/SCC component and did not occur in SCLC component, "increased in LCC/ADC/SCC" was defined as mutations that did not belong to "clonal both", "subclonal both" and "novel clonal in LCC/ADC/SCC", and mutations CCF in LCC/ADC/SCC were greater than in SCLC. Same definition as in SCLC. "non-selective" included "clonal both" and "subclonal both". "positive selection in LCC/ADC/SCC" included "novel subclonal in LCC/ADC/SCC”, "novel clonal in LCC/ADC/SCC”, and "increased in LCC/ADC/SCC”. “positive selection in SCLC” included “novel subclonal in SCLC", “novel clonal in SCLC", and “increased in SCLC".

Measures of evolutionary pressures of two components for each patient were calculated by dN/dS ratio on protein-coding regions. Intuitive interpretation of dN/dS<1 as negative selection, dN/dS = 1 as neutrality, and dN/dS>1 as positive selection.

***Timing mutations***

The timing of SNVs was determined by EstimateClonality (v1.0). Briefly, we estimated the cellular prevalence of somatic mutations based on tumor purity and CNV and mutation copy number. Early mutations were defined as a mutation copy number of >1, whereas, late ones were classified as a mutation copy number of < = 1.

***Differential expression gene and pathway enrichment***

We performed differential expression gene analysis of SCLC components and other non-small cell components using *DESeq2* R package in samples with SCLC combined LCC and sample with SCLC combined ADC/SCC respectively. Genes which adjust p value less than 0.05 and |log2(fold change)|>1 were considered statistically differential. SCLC components were used as control and non-small cell components as case. The Gene Set Enrichment Analysis and visualization were analyzed by *fgsea* R package based on preranked genes by all log2FC values between groups. Pathways with Adjust p value less than 0.05 and absolute value of normalized enrichment score greater than 1 were considered to be significant enrichment.

***Immune cell infiltration and immunity***

We used single-sample Gene Set Enrichment Analysis (ssGSEA) method of GSVA R package to quantify the relative infiltration of 28 immune cell types in the tumor microenvironment. Marker genes for each immune cell type were obtained from a study, the parameter settings were as follows: *method='ssgsea'*, *kcdf='Gaussian'*, and *abs.ranking=TRUE*. An enrichment score obtained by ssGSEA analysis represented the relative infiltration of each immune cell type. The enrichment score was normalized to unity distribution, for which zero is the minimal and one is the maximal score for each immune cell type.

We also used GSVA R package to estimate immunity based on 25 innate and adaptive immune related signatures reported in previous paper with *method=’gsva'* and *mx.diff=FALSE* parameter settings . Finally, each sample had an enrichment score for each immune signature.

The immune microenvironment score, immune score, and stroma score were estimated by ESTIMATE R package.

***Immune infiltration classification***

We used ConsensusClusterPlus R package with k-means clustering algorithm and Euclidean distance measurement to select optimal clustering result and determine the stability and consistency of immune infiltration clustering results. The immune infiltration clustering result was displayed by ComplexHeatmap R package.

***HLA genotyping and HLALOH identification***

HLA genotyping was predicted by OptiType which was an HLA genotyping algorithm based on integer linear programming, capable of producing accurate 4-digit HLA genotyping predictions from NGS data by simultaneously selecting all minor and major HLA-I alleles.

HLALOH (Loss Of Heterozygosity in Human Leukocyte Antigen) was identified by HLALOH repository, a computational tool to evaluate HLA loss using next-generation sequencing data and HLA genotyping .

***Neoantigen identification***

We used pVACseq software to predict MHC-I class neoantigen based on mutations which could be missense, in-frame insertion, in-frame deletion, protein-altering, and frameshift mutations and HLA genotyping. MHC-I class prediction Algorithm included NetMHC, NetMHCpan, PickPocket, SMM, and SMMPMBEC modules. Filtering variants was processed by IEDB by read coverage and mutation VAF with parameters setting: *--normal-vaf 0.02 --tdna-vaf 0.10 --tdna-cov 10 --trna-vaf 0 --trna-cov 0 --expn-val 0 --maximum-transcript-support-level 1*.

***Tumor Inflammation Signature (TIS) score***

The TIS score measured adaptive immune response within tumors based on 18-gene as described in Patrick Danaher's study. The TIS score was calculated as the mean expression of 18 genes.

***Relationship between Immune distance and genomic distance***

The immune distance between two components of each patient was calculated based on immune cell infiltration by the Euclidean distance method. The genomic distance between two components of each patient was calculated based on all mutations by the Euclidean distance method. All mutations present in any component from a patient were turned into a binary presence/absence matrix, where the rows were mutations and columns tumor components. Pearson was used to calculate the correlation between immune distance and genomic distance of cSCLC.

***Neoantigen depletion***

A neoantigen was considered to be expressed if at least four RNA-seq reads mapped to the mutation position, and at least two contained the mutated base. Neoantigen depletion was identified with transcriptional neoantigen depletion and copy number neoantigen depletion. Specifically, non-synonymous mutations which were predicted to be neoantigens were not expressed or occurred copy number loss. A fisher's test was performed to determine if non-synonymous mutations that were neoantigens were less likely to be expressed or in regions of copy number loss as compared to the non-synonymous mutations which were not predicted to be neoantigens. Odd ratios <1 indicate that putative neoantigens are less likely to be expressed, as compared to non-synonymous mutations that are not putative neoantigens. Odd ratios >1 indicate neoantigens are more likely to be in regions of copy-number loss than non-synonymous mutations that are not neoantigens.

***Immunoediting score***

$$immunoediting score=\frac{B_{\mathrm{obs}}/N_{\mathrm{obs}}}{B_{\mathrm{pred}}/N_{\mathrm{pred}}}$$

Where *N_obs_* is the observed number of missense mutations and *B_obs_* the predicted number of neoepitopes for each sample, *N_pred_* is the expected number of missense mutations and *B_pred_* the expected number of neoepitopes were calculated for each sample.

This method compares the observed and expected numbers of neoantigens present in a tumor, such that a score of < 1 suggests DNA immunoediting has occurred.

***Immune evasion capacity***

High immune evasion capacity of tumor had middle or low level of immune infiltration and antigen presenting dysfunction or DNA immunoediting score less than 1. Conversely, low immune evasion capacity of tumor had high immune infiltration or no evidence of immune evasion (DNA immunoediting score > 1 and no antigen presentation disruption).

***Pervasive disruption to antigen presentation***

Non-synonymous mutations or copy number loss of antigen-presenting pathway genes affected the HLA enhanceosome, peptide generation, chaperones, or the MHC complex itself, these genes included CIITA, IRF1, PSME1, PSME2, PSME3, ERAP1, ERAP2, HSPA, HSPC, TAP1, TAP2, TAPBP, CALR, CNX, PDIA3, B2M.

***Calculation of the fraction of lung epithelial cells of bulk RNA-seq data***

We used single-cell data of normal lung tissue [24] to create a signature matrix for discriminate each cell types and applied the signature matrix to bulk RNA profiles to infer cell type proportions. The above processes were performed by CIBERSORTx [25].

***Transformation direction of cSCLC***

We inferred the transformation direction between the two components in SCLC combined with ADC or SCC by integrating the four dimensions of tumor location, driver gene clonality, lung epithelial cell fraction, and subclonal structure of each cSCLC sample. We identified two transformation modes in cSCLC. The transformation from ADC to SCLC in each cSCLC tumor met the following conditions: 1) AT2 cells or AT1 accounted for the highest proportion in ADC tumor samples, and PNEC accounted for the highest proportion in SCLC samples ; 2) The tumor location of cSCLC is peripheral; 3) EGFR mutation is the trunk clonal event or RB1 is a unique event in SCLC; 4) The subclonal structure satisfies the clonal evolution from ADC to SCLC.

The transformation direction of SCLC to SCC met the following conditions: 1) SCLC has the highest proportion of PNEC, while SCC has the highest proportion of Basal cells; 2) The tumor location of such cSCLC is usually central; 3) TP53 and RB1 non-synonymous mutations and copy number loss events were trunk clonal events , EGFR or KRAS are more specific in SCC; 4) The subclonal structure satisfies the clonal evolution from SCLC to SCC.

***Statistics analysis***

The Mann Whitney's paired test was used for paired sample comparison. Mann-Whitney test was used to compare the differences between two groups. Correlation analysis was performed by Pearson's correlation coefficient for mutSigCV genes distribution of combined tumor and pure tumor. A p-value less than 0.05 was considered statistically significant. Hierarchical cluster analysis was used to cluster mutation spectrum of different tumor types.

22. Burrell RA, Burrell, R. A., McClelland, S. E., Endesfelder, D., Groth, P., Weller, M. C., Shaikh, N., Domingo, E., Kanu, N., Dewhurst, S. M., Gronroos, E., Chew, S. K., Rowan, A. J., Schenk, A., Sheffer, M., Howell, M., Kschischo, M., Behrens, A., Helleday, T., Bartek, J., Tomlinson, I. P., Swanton, C. Replication stress links structural and numerical cancer chromosomal instability. Nature 2013;494:492-496.

23. Leshchiner, I., Livitz, D. G., Gainor, J. F., Rosebrock, D., Spiro, O., Martinez, A., Mroz, E. A., Lin, J. J., Stewart, C., Kim, J., Elagina, L., Mino‐Kenudson, M., Rooney, M., Ignatius Ou, S.-H., Wu, C. J., Rocco, J. W., Engelman, J. A., Shaw, A. T., Getz, G. J. b. Comprehensive analysis of tumour initiation, spatial and temporal progression under multiple lines of treatment. bioRxiv508127(2019)doi:10.1101/508127.

24. Travaglini, K. J., Nabhan, A. N., Penland, L., Sinha, R., Gillich, A., Sit, R. V., Chang, S., Conley, S. D., Mori, Y., Seita, J., Berry, G. J., Shrager, J. B., Metzger, R. J., Kuo, C. S., Neff, N., Weissman, I. L., Quake, S. R., Krasnow, M. A. A molecular cell atlas of the human lung from single-cell RNA sequencing. Nature. 2020; 587, 619-625.

25. Newman, A. M., Steen, C. B., Liu, C. L., Gentles, A. J., Chaudhuri, A. A., Scherer, F., Khodadoust, M. S., Esfahani, M. S., Luca, B. A., Steiner, D., Diehn, M., Alizadeh, A. A. Determining cell type abundance and expression from bulk tissues with digital cytometry. Nat Biotechnol 2019;37: 773-782.

**Additional Supplementary Tables in Excels Files:**

**Supplementary Table 1.** Clinical details of cSCLC cases.

**Supplementary Table 2.** All somatic mutations of cSCLC samples.

**Supplementary Table 3.** Potential driver genes in cSCLC samples.

**Supplementary Table 4.** Other recurrently mutant genes in cSCLC samples.

**Supplementary Table 5.** Predicted neoantigens in cSCLC samples.

**Supplementary Table 6.** Somatic copy number variations in cSCLC samples.

**Supplementary Table** **7.** Significant broad copy number variations in cSCLC samples.

**Supplementary Table 8.** Significant focal copy number variations in cSCLC samples.

**Supplementary Table 9.** CCF and clone clusters of somatic non-synonymous mutations in cSCLC samples.

**Supplementary Table 10.** clonality of somatic non-synonymous mutations in cSCLC samples.

**Supplementary Table 11.** Somatic evolutionary timings of mutations.

**Supplementary Table 12.** Immune cell fraction of cSCLC samples.

**Supplementary Table 13.** Immune-related pathways enrichment score of cSCLC samples.

**Supplementary Figures:**

**Supplementary Figure 1.** An overview of the design of this study. A) Schematic diagram of study design; B) Schematic diagram of laser microdissection, left picture represents the IHC staining of cSCLCs before laser microdissection, the right represents the IHC staining of cSCLCs after laser microdissection, the yellow dotted line represents the lung adenocarcinoma component to be separated.


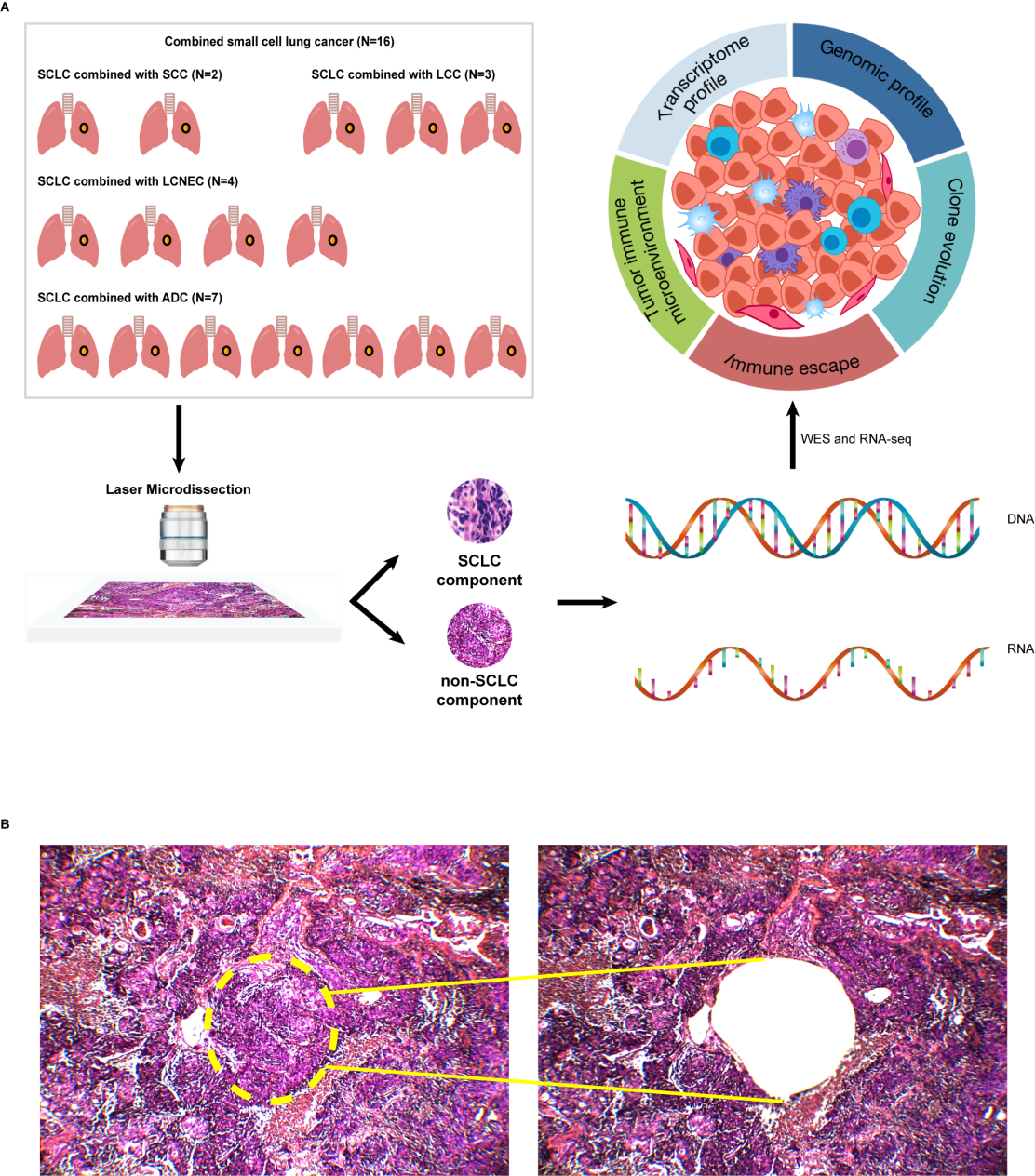


**Supplementary Figure 2.** Genomic biomakers in cSCLCs. **A**) Distribution of the 96 mutation types combined across cSCLCs; Mutational signatures B) between LCC and paired SCLC components; C) between ADC/SCC and paired SCLC components; D) between different tumor types; E) between L-SCLC and AS-SCLC; Boxplot in TMB F) and TNB G) between L-SCLC and AS-SCLC; Boxplot in TMB H) and TNB I) at the tumor types level; wGII comparisions J) at the tumor types level, and K) between L-SCLC and AS-SCLC. The percentage of occurred whole genome doubling L) between LCC and paired SCLC components; M) between ADC/SCC and paired SCLC components.


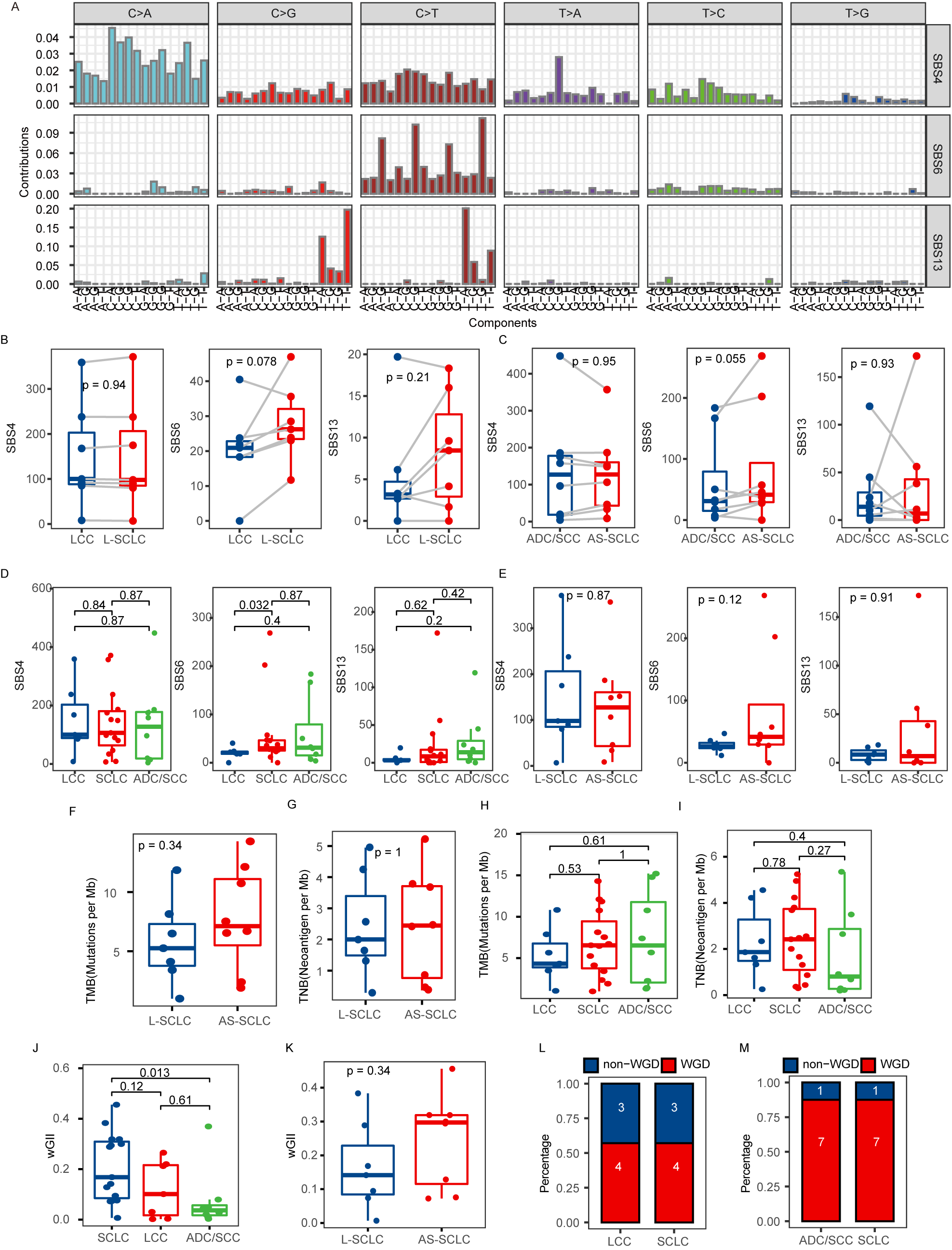


**Supplementary Figure 3.** Copy number variations profile of cSCLCs. Frequencies of copy number variations in chromosome arm-level of A) SCLC and paired ADC/SCC, B) SCLC and paired LCC. Recurrent focal CNVs in C) SCLC and paired LCC, D) SCLC and paired ADC/SCC, with potential CNV drivers annotated on the circos plot. Focal amplications were marked by red color, Focal deletions were marked by blue color.


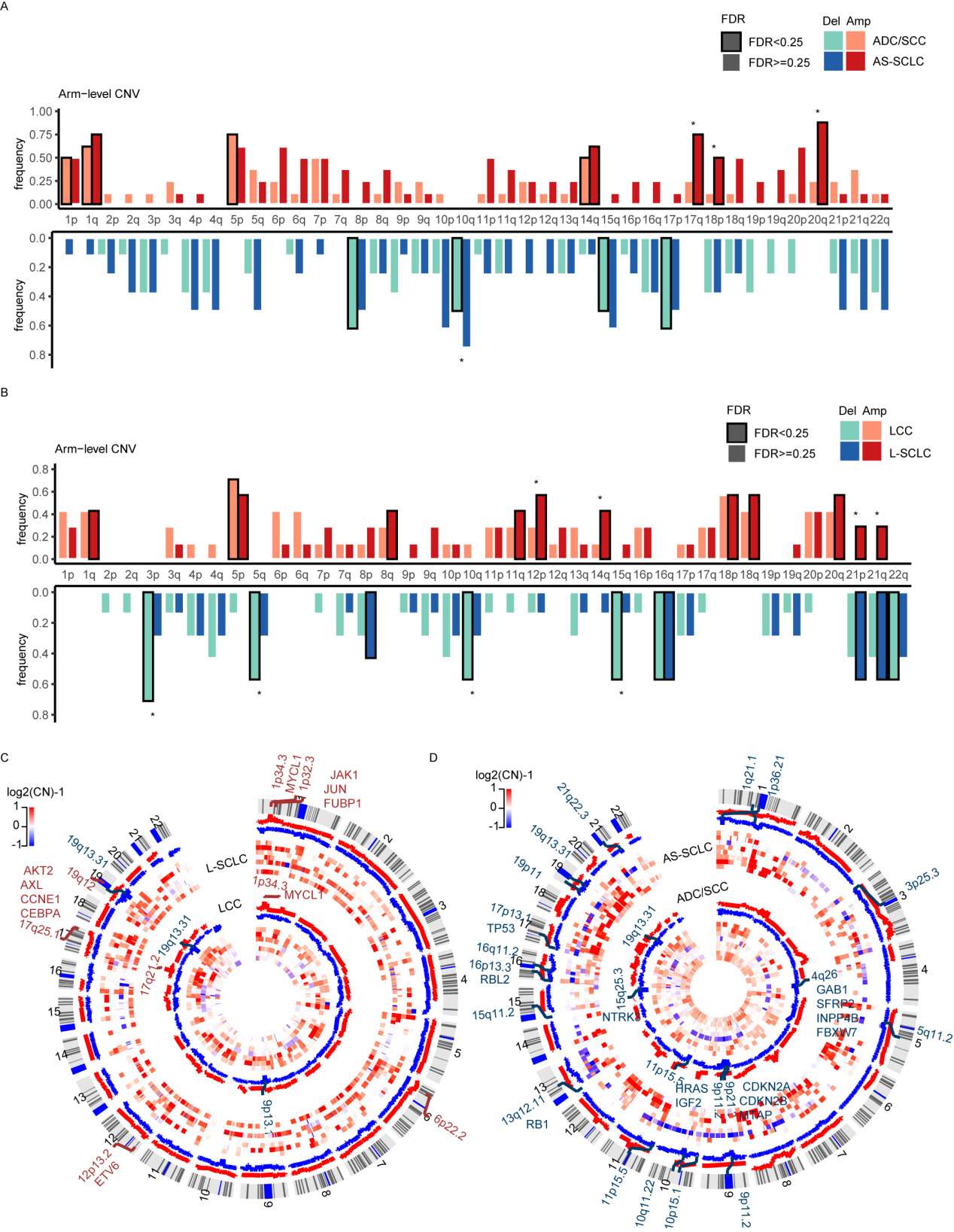


**Supplementary Figure 4.** Driver genes between pure tumors and cSCLCs. A) The MutSigCV p-value of drivers A) between LCC components and pure LCC tumors; B) between ADC/SCC components and pure LUAD/LUSC tumors; C) between SCLC components and pure SCLC tumors; D) between LCC components and paried SCLC components; E) between ADC/SCC components and paired SCLC tumors. F) The tumor mutation burden between pure tumors and cSCLC. The frequency of G) TP53, H) RB1, I) EGFR between pure tumors and cSCLC. J) Hierarchical clustering based on mutational spectrum on cSCLC and the corresponding pure tumors. K) SCLC subtypes in SCLC components and pure SCLC tumors.

**
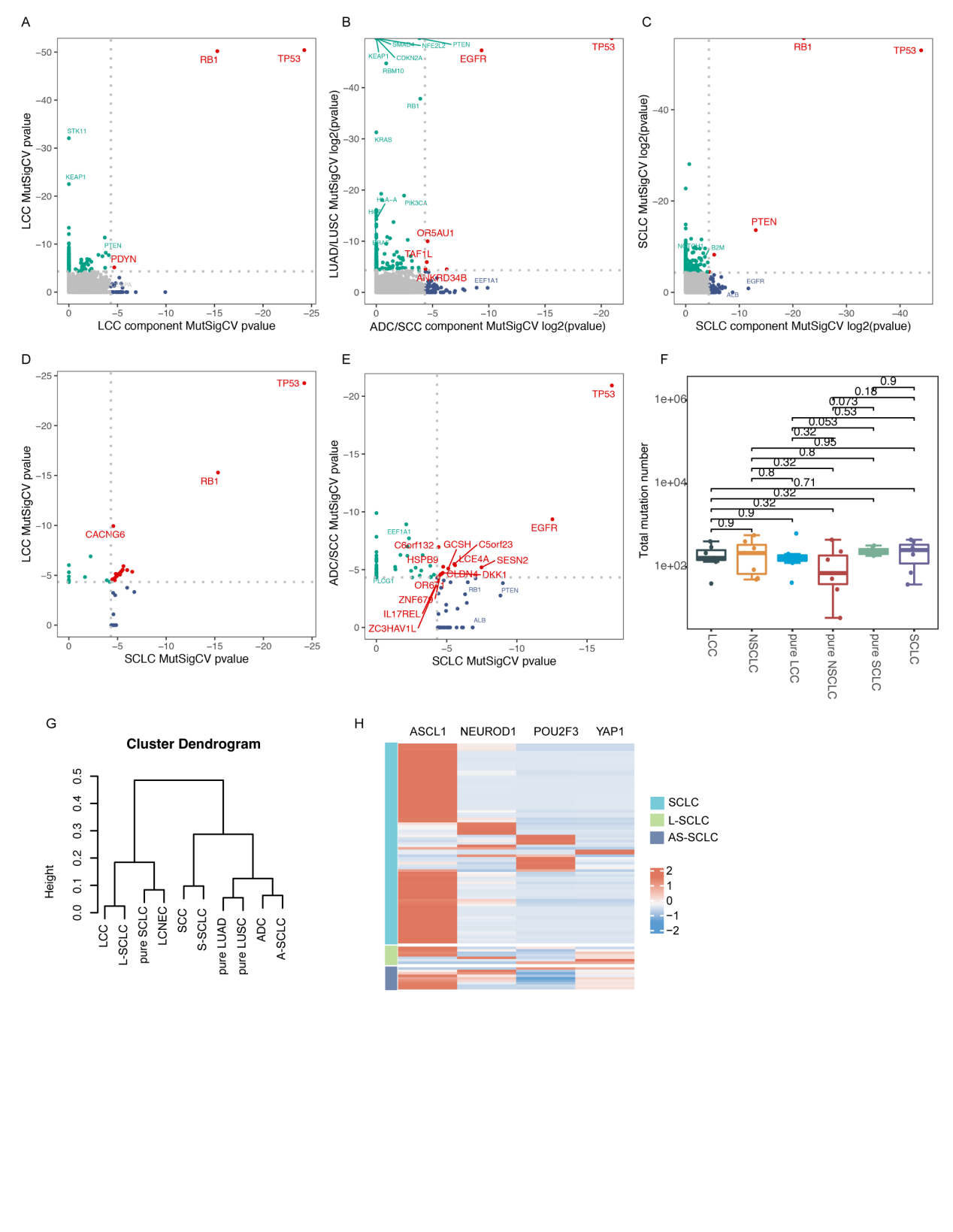
**

**Supplementary Figure 5.** Enrichment of Immune-related pathways and immune cell infiltration in CSCLCs. The comparisions of A) immune cell infiltration, B) tumor immune microenvironment, and C) TIS between L-SCLC and AS-SCLC components; D) The enrichment score heatmap of immune-related pathways in CSCLCs, and statistics p-value between different components; E) The comparisions of enrichment scores of immune-related pathways between L-SCLC and AS-SCLC components.


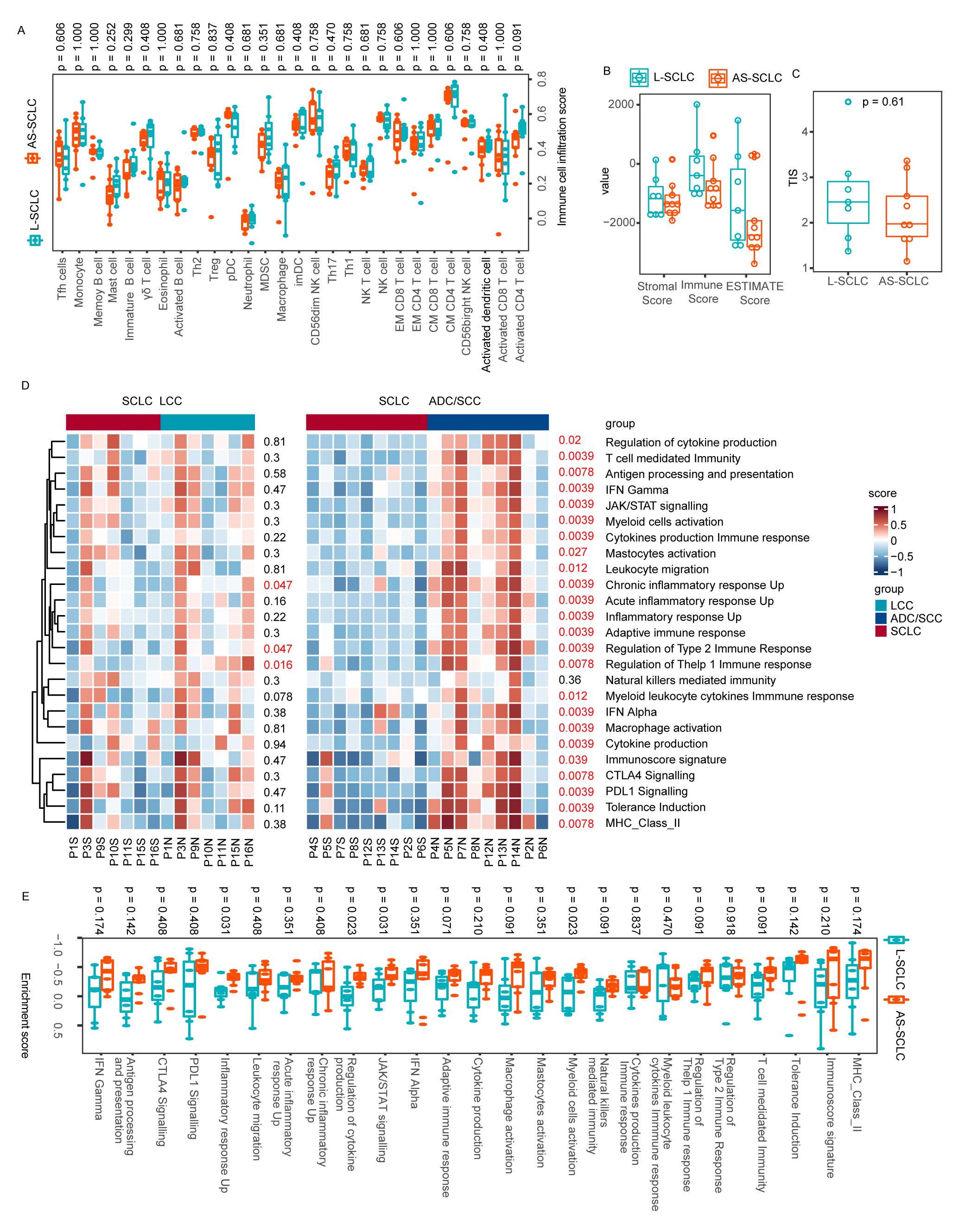


**Supplementary Figure 6.** Clonality in CSCLCs. The bar plots showed clonal types of each driver genes in different components of A) SCLC-LCC subtypes and B) SCLC-ADC/SCC subtypes; C) The distribution of somatic mutations clonality between different components in each cSCLC tumor; The comparisions of D) no selection, E) positive selection in non-SCLC components, and F) positive selection in SCLC components between SCLC-LCC subtypes and SCLC-ADC/SCC subtypes; The assocations between G) tumor purity, H) LCC/SCC tumor proporition, I) ADC/SCC/SCLC tumor proporition, J) tumor stages of non-SCLC components, K) tumor stages of SCLC components and the ccorresponding subclonal ratios.


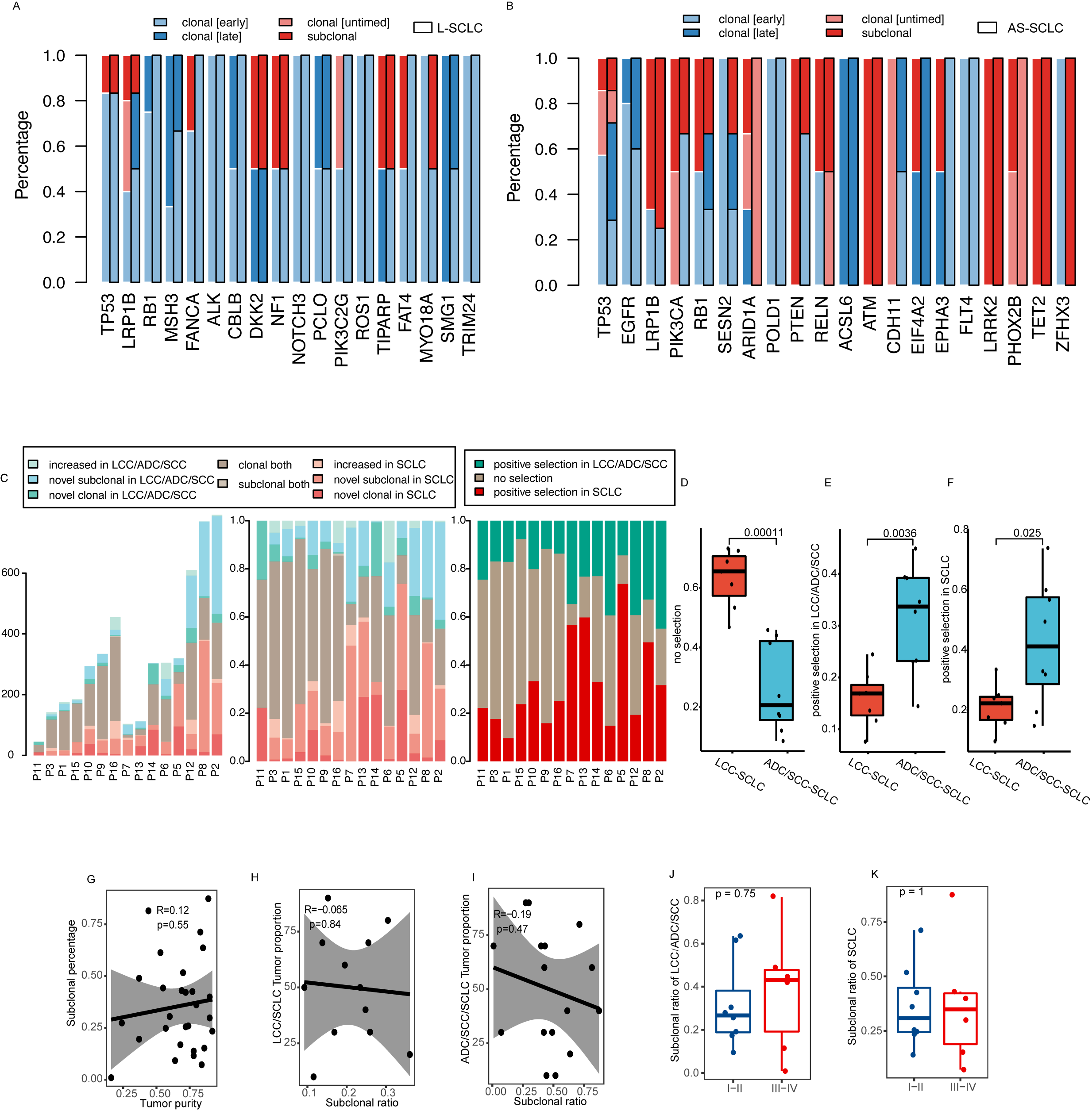


**Supplementary Figure 7.** Epithelial cell components of cSCLCs and tranformation modes in CSCLCs. A) the proportion of epithelial cell components in cSCLCs; B) subclonal structure of patients which are transformed from ADCs to SCLCs; C) subclonal structure of patients which are transformed from SCLCs to SCCs; D) The evolutionary fishplot of P12 patient; E) 3D reconstruction of P14's CT scan; F) IHC staining of primary tumor and recurrent tumor of P14. G) subclonal structure of patients which cannot be inferred the transformation direction.


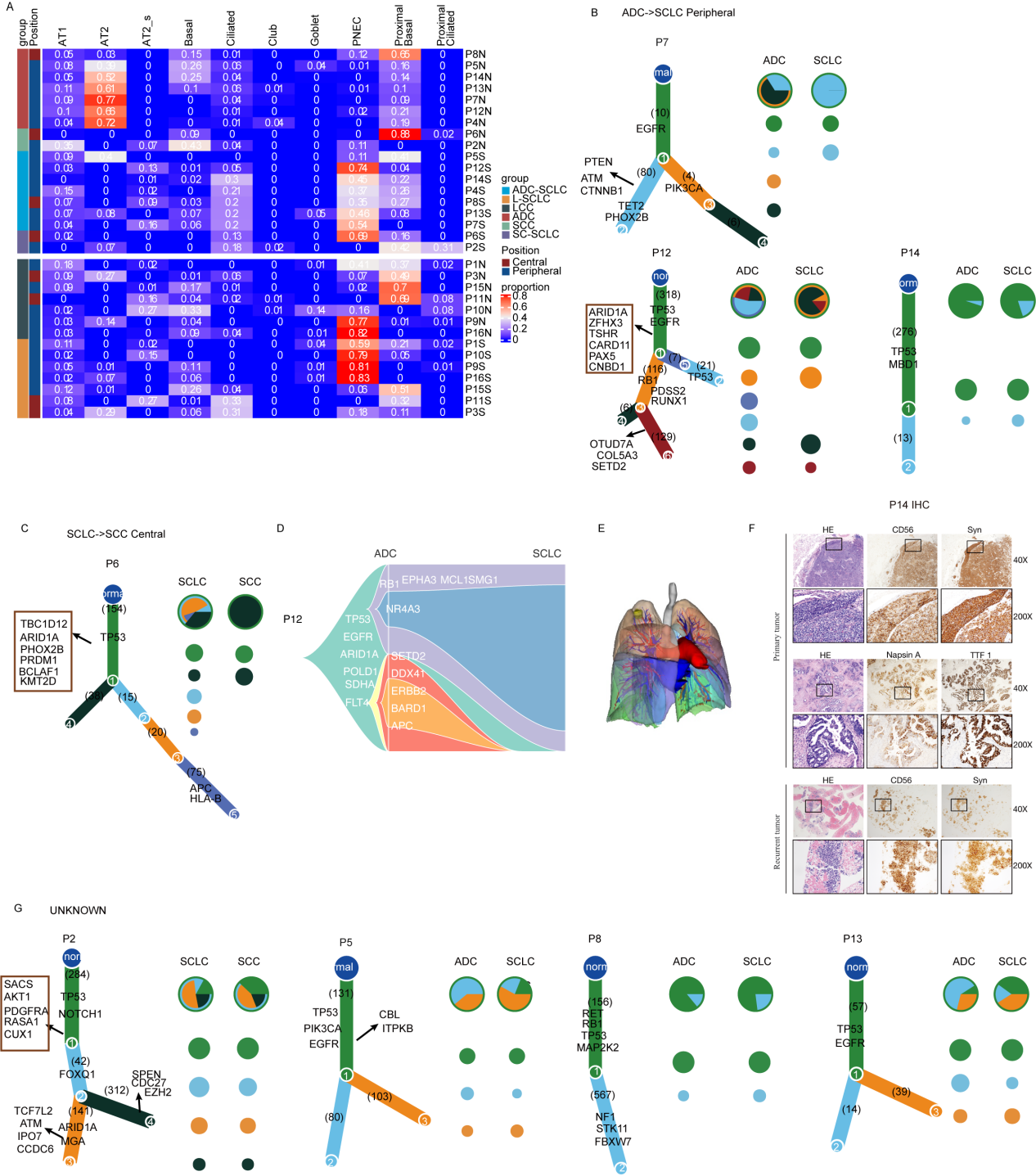


**Supplementary Figure 8.** Neoantigen depletion in CSCLCs. The odds ratio to occur neoantigen expressed in A) different tumor subtypes and B) different levels immune cell infiltration. The odds ratio to generate neoantigen in consistently expressed genes C) different tumor subtypes and D) different levels immune cell infiltration. The odds ratio to neoantigen occurred in all copy number loss region in E) different tumor subtypes and F) different levels immune cell infiltration. The odds ratio to neoantigen occurred in clonal copy number loss region in G) different tumor subtypes and H) different levels immune cell infiltration. The likely to neoantigen occurred in subclonal copy number loss region in I) different tumor subtypes and J) different levels immune cell infiltration.

**
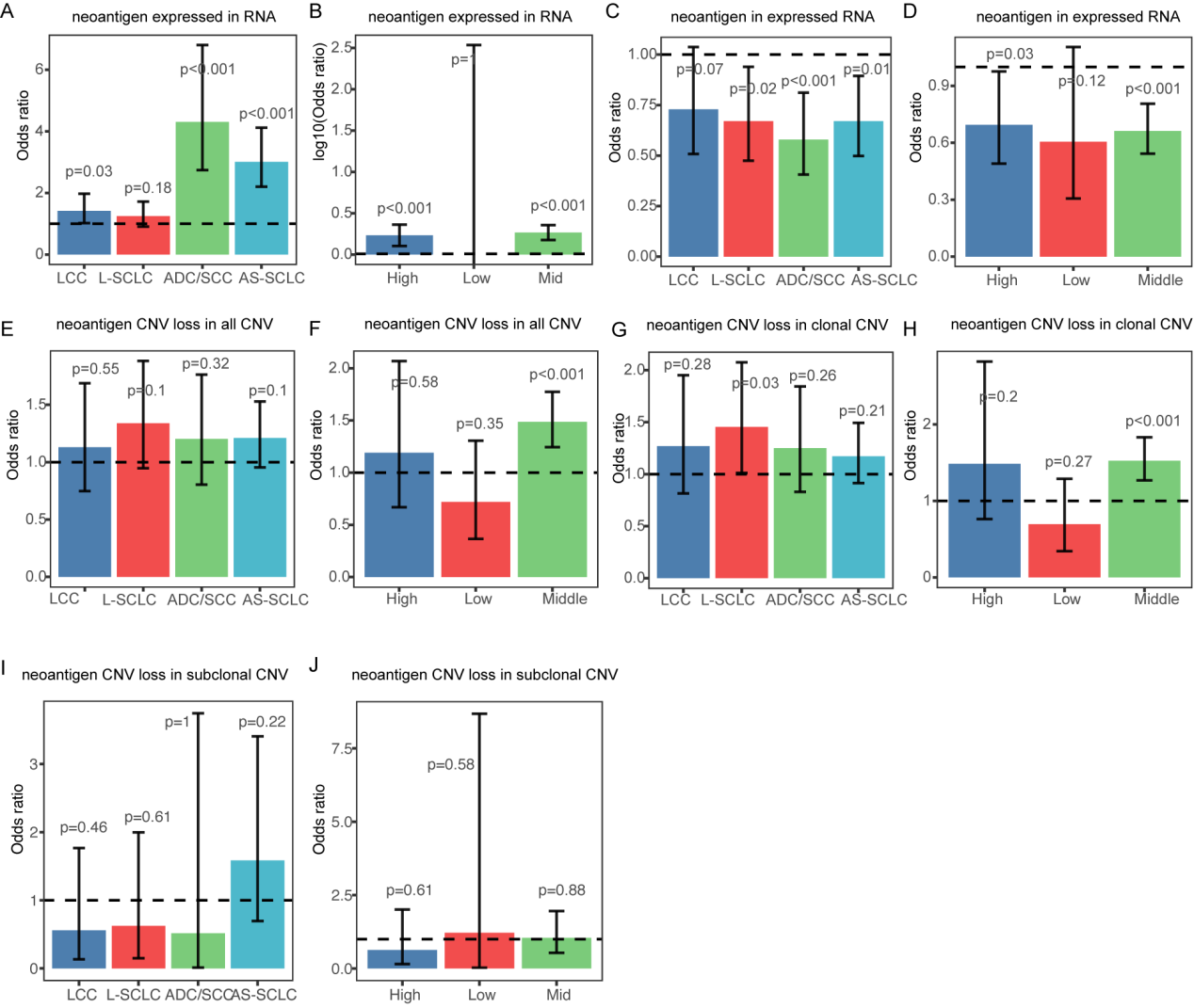
**

**Supplementary Figure 9.** The associates between RB1 and genomic biomarkers in cSCLCs. A) The correlation between immune distance and the pairwise genomic distance. The weighted chromosome instability comparisons between different TP53 and RB1 alterations status B) in the whole cohort and C) SCLC-ADC/SCC cohort. Comparisons of the whole genome doubling between different TP53 and RB1 alterations status D) in the whole cohort and E) SCLC-ADC/SCC cohort. H) DDR pathways alterations between SCLC components and LCC/ADC/SCC components. I) Differently expressed genes of DDR pathways in SCLC versus ADC/SCC. J) The enrichment score of DDR pathways among tumors with different alteration status of TP53 and RB1.


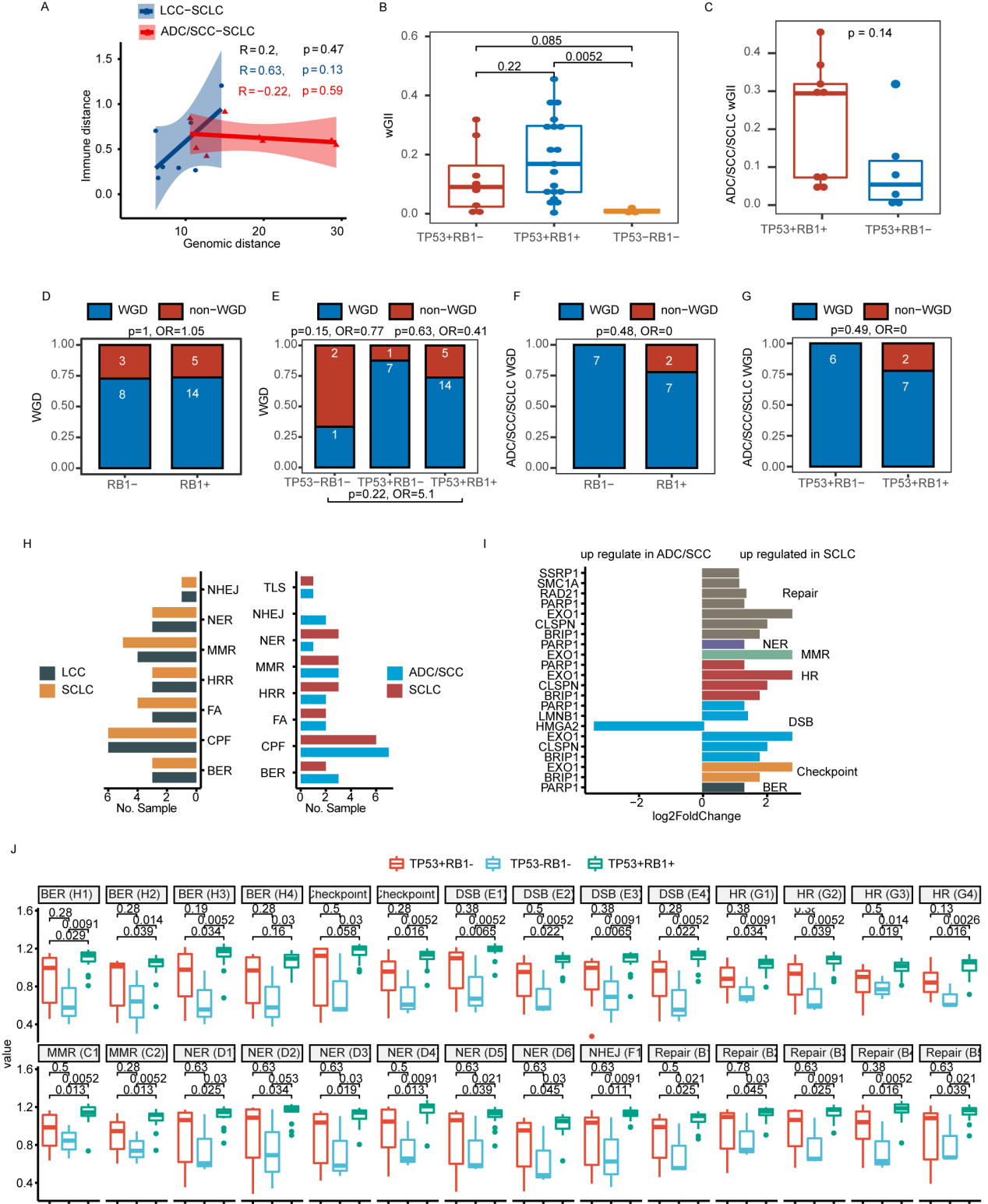

Supplement: Supplementary file 1 — Additional file 1.. Table S1. Clinical details of cSCLC cases. Table S2. All somatic mutations of cSCLC samples. Table S3. Potential driver genes in cSCLC samples. Table S4. Other recurrently mutant genes in cSCLC samples. Table S5. Predicted neoantigens in cSCLC samples. Table S6. Somatic copy number variations in cSCLC samples. Table S7. Significant broad copy number variations in cSCLC samples. Table S8. Significant focal copy number variations in cSCLC samples. Table S9. CCF and clone clusters of somatic non-synonymous mutations in cSCLC samples. Table S10. clonality of somatic non-synonymous mutations in cSCLC samples. Table S11. Somatic evolutionary timings of mutations. Table S12. Immune cell fraction of cSCLC samples. Table S13. Immune-related pathways enrichment score of cSCLC samples. [file 12967_2024_4968_MOESM1_ESM.docx]
